# Uncovering diversity and abundance patterns of CO_2_-fixing microorganisms in peatlands

**DOI:** 10.1038/s44185-025-00099-1

**Published:** 2025-08-04

**Authors:** Marie Le Geay, Kyle Mayers, Anna Sytiuk, Ellen Dorrepaal, Martin Küttim, Mariusz Lamentowicz, Eeva-Stiina Tuittila, Béatrice Lauga, Vincent E. J. Jassey

**Affiliations:** 1 Université de Toulouse, Toulouse INP, CNRS, IRD, CRBE, Toulouse, France; 2 Molecular Ecology and Paleogenomics - MEP, NORCE Research, Bergen, Norway; 3 Climate Impacts Research Centre, Department of Ecology and Environmental Science, Umeå University, Abisko, Sweden; 4 Institute of Ecology, School of Natural Sciences and Health, Tallinn University, Tallinn, Estonia; 5 Climate Change Ecology Research Unit, Adam Mickiewicz University, Poznań, Poland; 6 School of Forest Sciences, Joensuu campus, University of Eastern Finland, Joensuu, Finland; 7 Universite de Pau et des Pays de l’Adour, CNRS, IPREM, Pau, France

**Keywords:** Community ecology, Microbial ecology, Molecular ecology

## Abstract

Microorganisms play a crucial role in the carbon (C) dynamics of peatlands — a major terrestrial C reservoir. Because of their role in C emissions, heterotrophic microorganisms have attracted much attention over the past decades. CO_2_-fixing microorganisms (CFMs) remained largely overlooked, while they could attenuate C emissions. Here, we use metabarcoding and digital droplet PCR to survey microorganisms that potentially fix CO_2_ in different peatlands. We demonstrate that CFMs are abundant and diverse in peatlands, with on average 1021 CFMs contributing up to 40% of the total bacterial abundance. Using a joint-species distribution model, we identified a core and a specific CFM microbiome, the latter being influenced by temperature and nutrients. Our findings highlight that ASV richness and community structure were direct drivers of CFM abundance, while environmental parameters were indirect drivers. These results provide the basis for a better understanding of the role of CFMs in peatland C cycle inputs.

## Introduction

Peatlands are large terrestrial carbon (C) sink, storing about 30% of all soil C (500–1000 Gt of C) for only 3% of land area^1^. This storage of C hinges on the imbalance between C uptake through photosynthesis and C loss through respiration and decomposition. For peatlands at high latitudes, cold temperature, waterlogged conditions and poor nutrient availability^2^ preserve organic matter from decomposition, making peatlands an essential factor in the climate system^3^. Microbial growth and activity are important players of the peatland C dynamics as microorganisms are involved in key processes of the peatland C cycle, such as decomposition, respiration, methanogenesis and CO_2_ fixation^4,5^. Because climate warming could accelerate the microbially driven efflux of CO_2_ toward the atmosphere^6^, heterotrophic microorganisms have received increasing attention over the last decades^4,7^ whilst CO_2_-fixing microorganisms (CFMs) remained overlooked. Yet, CFMs could attenuate, to some extent, microbial CO_2_ emissions in response to warming^8,9^.

CFMs can fix atmospheric CO_2_ through seven natural metabolic pathways including the Calvin-Benson-Bassham (CBB) cycle (reductive pentose phosphate cycle), the rTCA cycle (reductive citrate cycle), the 3-HP/4-HB cycle (3-hydroxypropionate/4-hydroxybutyrate cycle), the 3-HP cycle (3-hydroxypropionate bi-cycle), the Wood–Ljungdahl pathway (the reductive acetyl-CoA pathway), the DC/4-HB cycle (dicarboxylate hydroxybutyrate cycle) and the reductive glycine pathway^10–12^. Among these metabolic pathways, the CBB cycle was the first discovered and is the most widespread^13^. In particular, the CBB cycle is found in all oxygenic phototrophs, and in chemoautotrophs^14,15^. Oxygenic phototrophs fix CO_2_ by harnessing the energy of light, through photosynthesis while chemoautotrophs power the CBB cycle by oxidizing chemicals or molecular hydrogen^16^. Recent work further showed that some aerobic anoxygenic phototrophic bacteria (AAnPBs) possess and express CBB genes, suggesting they fix atmospheric CO_2_ too^17^. While the mechanisms of microbial CO_2_ fixation through the CBB cycle are well documented, the main microbial diversity and abundance associated with CFMs using the CBB cycle remain poorly known in soils, and especially in peatlands.

Oxygenic phototrophs can be abundant and diverse in peatlands^5^, living either in the pore water, in the water film on *Sphagnum* mosses or in the hyaline *Sphagnum* cells that store water^18^. They are sensitive to temperature, soil water content and light availability, as well as plant cover, dissolved organic carbon and pH^5,19^. On the contrary, chemoautotrophs and AAnPBs have been overlooked, while they can be as abundant as oxygenic phototrophs^20^. As oxygenic phototrophs are essential for the peatland C cycle^21^, and because chemoautotrophs and AAnPBs could play an important role too^20,22^, better understanding the diversity, community composition, abundance and sensitivity to environmental parameters of these CFMs is essential to improve our comprehension of the peatland C cycle.

In this study, we combined a metabarcoding approach with digital droplet PCR (ddPCR) to explore how the abundance, diversity and community structure of microorganisms presenting the potential to fix CO_2_ using the CBB pathway, hereafter the CFMs, vary across peatland types and depth. In particular, we aim to identify which environmental variables drive CFM attributes and their interplay. Because of the strong variations in abiotic factors with depth and between peatland types, we predict that (i) patterns of CFM diversity and community composition will vary between peatlands sites and that (ii) such changes are mainly driven by nutrients, acidity and temperature along with precipitation, as these parameters are strong drivers of microbial communities^14,23^. Given their sensitivity to light availability and oxygen content, we also hypothesize that (iii) oxygenic phototrophs and AAnPBs will prevail in the upper peat layers whereas chemoautotrophs will be favored in deeper layers where reduced compounds are more abundant^24^. Finally, following the recent findings of Le Geay et al.^20^ showing the simultaneous presence of oxygenic phototrophs, chemoautotrophs and AAnPBs in peat samples, we propose that (iv) these functional groups will contribute to the peatland C fixation together instead of only oxygenic phototrophs.

## Results

### Partitioning of peatland sites according to environmental parameters

**Fig. 1:**
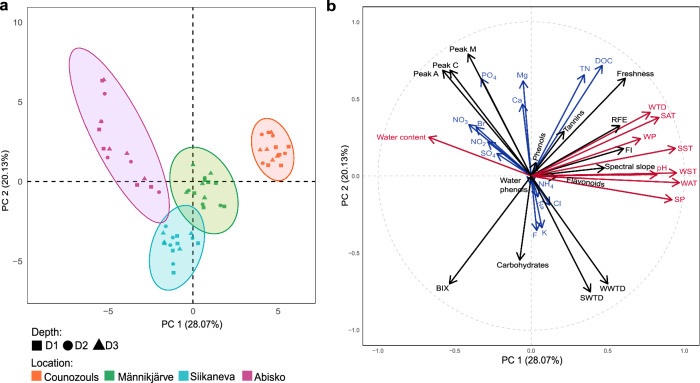
Principal component analysis (PCA) of all environmental variables. **a** Sample positioning in the PCA ordination space and **b** environmental variables contribution to PC axis 1 and 2. Black arrows represent dissolved organic matter quality, metabolites and water table depth variables, blue arrows and text highlight nutrient variables, and red arrows and text, variables related to climatic condition as well as pH. DOC dissolved organic carbon, TN total nitrogen, RFE relative fluorescence efficiency, BIX biological index, FI fluorescence index, SST spring soil temperature, SAT spring air temperature, SP spring precipitation, SWTD spring water table depth, WST winter soil temperature, WAT winter air temperature, WP winter precipitation and WWTD winter water table depth.

The four European peatland sites exhibited very different environmental conditions (Fig. 1; Supplementary Figs. 1–4; Supplementary Tables 1–3). The principal component analysis (PCA) revealed a net separation of peatland sites related to environmental parameters (Fig. 1a). The first axis separated samples between sites and was mainly driven by climatic conditions including soil temperature, air temperature, precipitations and pH (Fig. 1b). These environmental variables globally followed a gradient from south (Counozouls; highest values) to north (Abisko; lowest values; Supplementary Table 1; Supplementary Fig. 1). The second axis separating samples between sites and depths was mainly driven by nutrients (Fig. 1b). Some nutrient concentrations varied between sites (PO_4_^2-^ and Mg^2+^) while others varied with depth (K^+^, Br^-^ and F^-^; Supplementary Fig. 3). Metabolites and indexes of organic matter quality also contributed to both axis with few variations between sites and depths (Supplementary Figs. 2 and 4).

### Effects of depth and site on CFM abundances

**Fig. 2:**
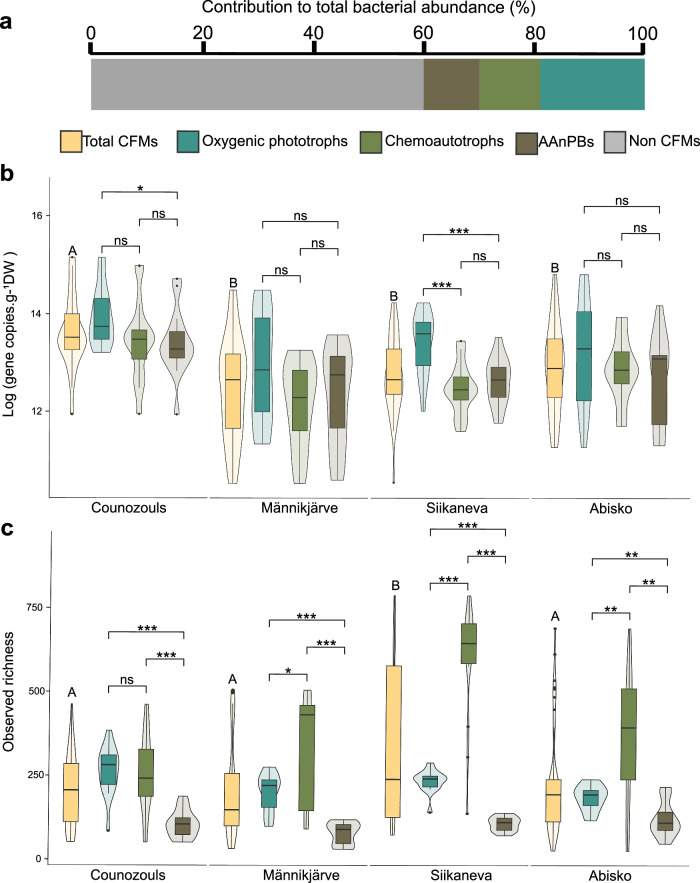
CFM abundance, richness and their response to environmental variables. **a** Average contribution of the different CFMs to total bacterial abundance (16S rRNA gene). **b** Absolute quantification using ddPCR, and **c** Observed richness of total CFMs (sum of 23S rRNA, *cbbL* and *pufM*/*bchYgenes*), oxygenic phototrophs (23S rRNA gene), chemoautotrophs (*cbbL*) and AAnPBs (*pufM/bchY*) for each site. Violin plots are showing the data distribution shape while boxplots are representing the logarithm of the total gene copies.g^−1^ DW. D1 = 0–5 cm; D2 = 5–10 cm and D3 = 10–15 cm. ns not significant; *: *P* < 0.05, **: *P* < 0.01 and ***: *P* < 0.001.

CFMs were highly abundant contributing up to 40% of the total bacterial abundance (Fig. 2a). The 23S rRNA, *cbbL* and *pufM* genes showed similar abundances, ranging from 7.89 × 10^4^ to 3.79 × 10^6^ copies.g^−1^ of dry peat for oxygenic phototrophs, from 3.74 × 10^4^ to 3.2 × 10^6^ copies.g^−1^ of dry peat for chemoautotrophs and from 5.31 × 10^4^ to 2.44 × 10^6^ copies.g^−1^ of dry peat for AAnPBs, respectively. Over all sites, we found a slight impact of location on CFM total abundance (sum of 23S rRNA, *cbbL* and *pufM* gene abundances; Fig. 2b, *P* < 0.05), whilst at the depth level, we did not find differences (Supplementary Fig. 5; Supplementary Tables 4 and 5).

The abundance of oxygenic phototrophs (23S rRNA gene) was very similar for the four sites and mostly decreased with depth (Supplementary Fig. 5b). Among oxygenic phototrophs, cyanobacteria represented ~50% of the abundance and decreased with depth (Supplementary Fig. 6). Chemoautotrophs abundance (*cbbL* gene) differed between sites with higher abundances in Counozouls and Abisko but did not vary much with depth (Supplementary Fig. 5c). AAnPB abundance (*pufM* gene) was also higher in Counozouls and Abisko and decreased with depth (Supplementary Fig. 5d).

### Effects of depth and site on CFM richness, diversity and community composition

In total, we found 7960 ASVs among which 2690 belonged to oxygenic phototrophs, 3879 ASVs belonged to chemoautotrophs and the remaining 1391 ASVs belonged to AAnPBs. The total observed richness of CFMs was relatively similar among sites, except Siikaneva which was slightly higher compared to other sites (*P* < 0.05; Fig. 2c; Supplementary Tables 6 and 7). This increase was driven by a strong increase in chemoautotrophic richness in Siikaneva when compared to other sites (Fig. 2c, *P* < 0.05). CFM richness also globally decreased with depth (Supplementary Fig. 7a–c). In terms of alpha-diversity (Shannon index), we found a few variations between sites (Supplementary Fig. 7d–f; Supplementary Tables 6 and 7). For oxygenic phototrophs, we found an increase in the alpha-diversity with depth (Supplementary Fig. 7d) while for chemoautotrophs and AAnPBs, alpha-diversity decreased with depth (Supplementary Fig. 7e, f). The non-metric multidimensional scaling (NMDS) analysis further confirmed that CFM communities were well structured both by site and depth (Supplementary Fig. 8). Chemoautotrophic and AAnPB communities of Siikaneva and Männikjärve were notably close (Supplementary Fig. 8b, c). Richness, together with alpha diversity and community structure, was very sensitive to environmental parameters, in particular nutrients, climate, RFE, and phenols content (Supplementary Fig. 7g).

**Fig. 3:**
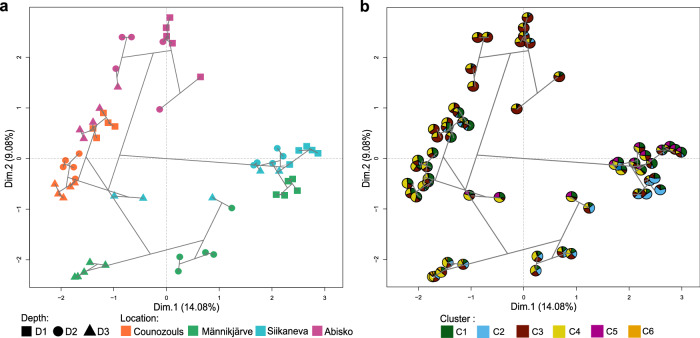
Multiple factor analysis (MFA) samples biplot. **a** MFA for 23S rRNA, *cbbL* and *bchY* genes ASV matrices. Geometric shapes represent each sample spilt according to depth and colors represent the four peatlands. **b** Pie charts represent the relative abundance of the different clusters (for the same samples) generated under the Joint Species Distribution Model (JSDM; see Fig. 5). Gray lines represent results of a hierarchical agglomerative clustering.

At the CFM community composition level, we merged the three ASV matrices (23S rRNA, *cbbL* and *bchY* genes) into a multiple factor analysis (MFA; Fig. 3). This multiple factor analysis emphasized clear patterns of community split between depth (first axis) and the four locations (second axis). In this MFA ordination space, community composition of Counozouls was closer to Abisko and community composition of Männikjärve was closer to Siikaneva (Fig. 3a). Among these communities of CFMs, we found an important diversity.

**Fig. 4:**
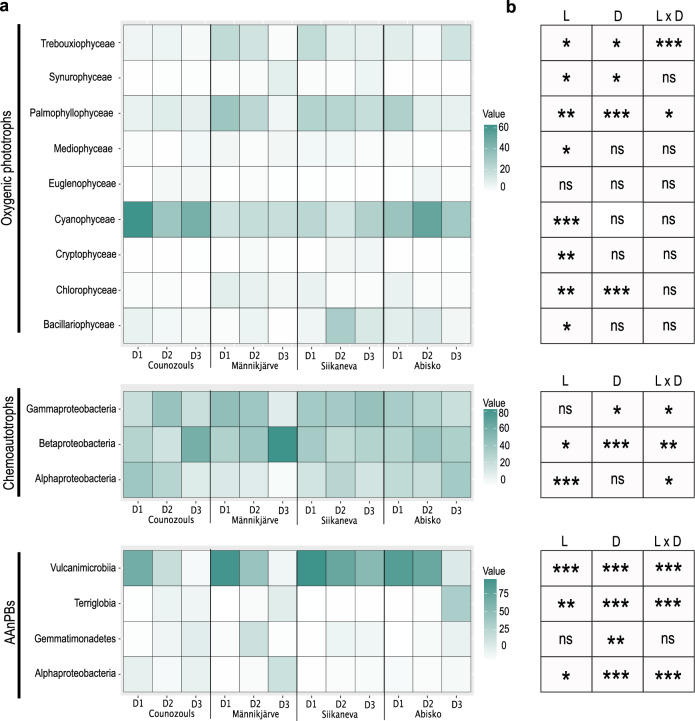
Impact of location and depth on relative abundance of ASVs aggregated by class. **a** Heatmaps showing the relative abundance (%) of ASVs aggregated by class according to location and depth. Only classes with abundance higher than 5% were kept. Light color represents low abundances while dark color represents higher abundances. **b** *P*-values from linear mixed effects models showing the effects of location, depth and location with depth on the relative abundance of each class. L location, D depth, ns not significant; *: *P* < 0.05, **: *P* < 0.01 and ***: *P* < 0.001; D1 = 0–5 cm; D2 = 5–10 cm and D3 = 10–15 cm.

Notably, oxygenic phototrophic ASVs belonged to 25 phyla, 41 classes, 76 orders and 102 families. These ASVs were dominated by Cyanophyceae followed by Palmophyllophyceae (Fig. 4a; Supplementary Figs. 9a and 10). Cyanophyceae were abundant in the four locations and at all depths (Fig. 4a). They were dominated by Nostocaceae and Chroococcidiopsidaceae families (Supplementary Fig. 9a). Palmophyllophyceae were mostly present in the surface samples of Männikjärve, Siikaneva and Abisko (Fig. 4a), and were dominated by Prasinococcaceae (Supplementary Fig. 9a). We also found that Trebouxiophyceae, Bacillariophycaea, Synurophycaea, and Chlorophyceae were abundant in the samples (Fig. 4a). Among oxygenic phototrophs we further found three species dominating the different sites, namely *Chroococcidiopsis sp*., *Prasinoderma coloniale* and *Anabaena sp*. Location, depth and location and depth together had a significant impact on oxygenic phototroph ASVs both at the class and at the family level (Fig. 4b; Supplementary Fig. 9b). However, these ASVs only represented 30% of the total 23S rRNA sequences generated, because 60% were not assigned using the *µgreen db algae* database.

Chemoautotroph ASVs belonged to 1 phylum, 3 classes, 7 orders and 13 families. Among chemoautotrophs, Proteobacteria, particularly Beta- and Gammaproteobacteria dominated all samples (Fig. 4a; Supplementary Figs. 9a and 11). Betaproteobacteria were notably abundant in Counozouls and Männikjärve deepest samples (Fig. 4a). Betaproteobacteria were represented by three main families, namely Thiobacillaceae, Gallionellaceae and Comamonadaceae (Supplementary Fig. 9a). Gammaproteobacteria were present at all sites in D1 and D2 samples (Fig. 4a) with Ectothiorhodospiraceae and Acidiferrobacteraceae being the main families present (Supplementary Fig. 9a). Betaproteobacteria were affected both by location and depth (*P* < 0.05), while Gammaproteobacteria were only affected by depth (*P* < 0.05; Fig. 4b). Among chemoautotrophs, we found *Thiobaccilus sp*., *Nitrobacter winogradsky*, *Sulfurifustis variabilis*, *Sulfuricaulis limicola* and *Hydrogenophaga sp*. being the most abundant species.

AAnPB ASVs were composed of 11 phyla, 20 classes, 28 orders and 35 families among which Terriglobia, Gemmatimonadetes, Alphaproteobacteria, and Vulcanimicrobiia were the most abundant classes with a net dominance of Vulcanimicrobiia for depth D1 and D2 (Fig. 4a; Supplementary Figs. 9a and 12). At the family level, the family Vulcanimicrobiaceae also dominated largely (Supplementary Fig. 9a). Vulcanimicrobiia were significantly affected by both location (*P* < 0.001) and depth (*P* < 0.001; Fig. 4b).

### CFM communities group in distinct clusters

**Fig. 5:**
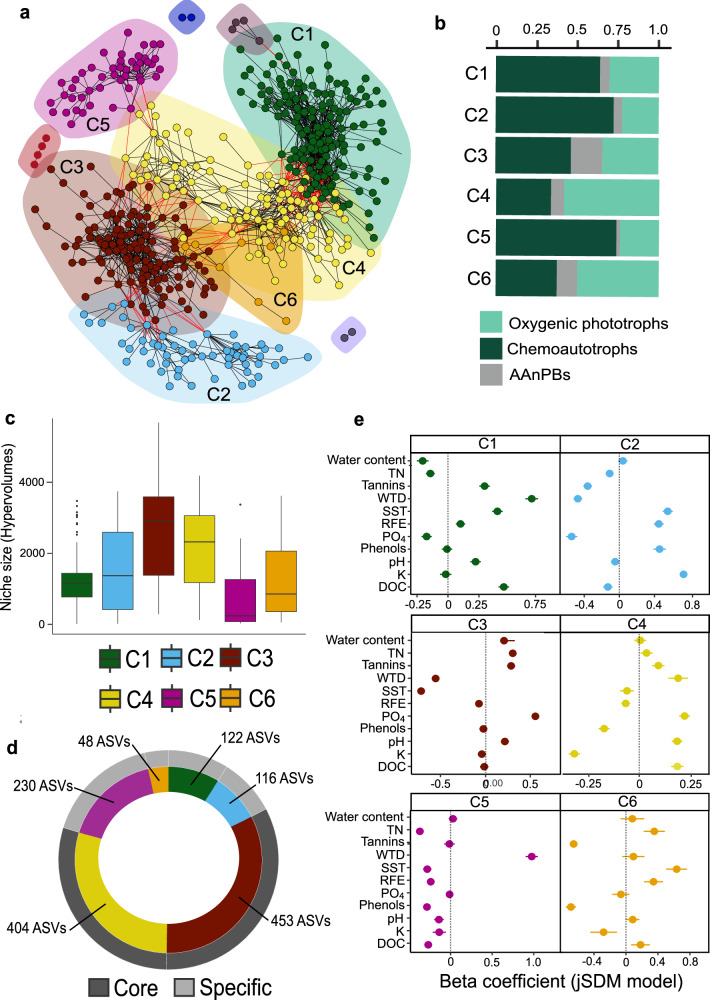
Co-occurrence network of the variation between ASVs caused by environmental parameters. **a** Clusters of co-occurring ASVs with each dot representing one ASV. **b** Barplot of the relative abundance of each microbial group within each cluster. **c** Niche size (hypervolumes) of each cluster. **d** Absolute distribution of ASVs into the six clusters and according if these ASVs are considered as core microbiome ASVs (dark gray) or specific microbiome ASVs (light gray). **e** Mean of beta coefficient from the JSDM model showing how environmental parameters are affecting the clusters. C1 cluster 1, C2 cluster 2, C3 cluster 3, C4 cluster 4, C5 cluster 5, C6 cluster 6, TN total nitrogen; DOC dissolved organic carbon; SST spring soil temperature; WTD water table depth; D1 = 0–5 cm; D2 = 5–10 cm and D3 = 10–15 cm.

Using Joint Species Distribution Modelling (JSDM), we identified six major clusters of CFMs with shared within-cluster responses, but opposite between-cluster responses, to environmental conditions (Fig. 5a). Each cluster was dominated by chemoautotrophs, except clusters 4 and 6 which were dominated by oxygenic phototrophs (Fig. 5b). Each cluster was also characterized by CFMs with different niche sizes (Fig. 5c). Clusters 3 and 4 exhibited ASVs with the largest niche size (Fig. 5c) and were logically widespread across all sites and depths (Fig. 3b). These clusters were both characterized by high abundances of oxygenic phototrophs (Fig. 5b), including Palmophyllocyceae, Cyanophyceae and Baccillariophyceae (Supplementary Figs. 13a and 14a), and chemoautotrophs (Fig. 5b) with Proteobacteria, mainly Acidiferrobacteraceae, Nitobacteraceae and Ectothiorhodospiraceae (Supplementary Figs. 13b and 14b). These clusters varied with environmental conditions, with cluster 3 related to cold and wet conditions and high concentrations of phosphorus and nitrogen whilst cluster 4 was driven by dry conditions and an environment rich in carbon and phosphorous (Fig. 5e).

Clusters 1, 2 and 6 showed intermediate niche sizes (Fig. 5c). They were accordingly not present in all sites and depths (Fig. 3b). Clusters 1 and 2 were dominated by chemoautotrophs (Fig. 5b) with a lot of Alpha and Betaproteobacteria among which Nitrobacteraceae, Thiobacillaceae and Comamonadaceae dominated (Supplementary Figs. 13b and 14b), followed by oxygenic phototrophs (Fig. 5b) composed of diverse classes and families (Supplementary Figs. 13a and 14a). Cluster 6 was composed of unclassified chemoautotrophs and Beta and Gammaproteobacteria (Supplementary Figs. 13 and 14). Clusters 1 and 6 were associated with warm, C and N-rich conditions. Cluster 2 was associated with wet and warm conditions, as well as with dissolved organic matter rich in phenols and samples rich in potassium (Fig. 5e). Cluster 5 had the lowest hypervolumes (Fig. 5c) and was present only in a few samples (Fig. 3b), mainly in Siikaneva. This cluster was dominated by chemoautotrophs (Fig. 5b) with Betaproteobacteria (Thiobacillaceae and Comemonadaceae), and by some specific oxygenic phototrophs (Supplementary Figs. 13a and 14a), and was strongly related to dry and nutrient-poor conditions (Fig. 5e).

Following the results of niche size we further classified ASVs belonging to clusters 3 and 4 as the core microbiome, since they were present in all our samples (Fig. 5d), and ASVs belonging to clusters 1, 2, 5, and 6 as the specific ASVs, only present in a few sites. Clusters 3 and 4 shared 62% of the ASVs (453 ASVs – 33% for C3 and 404 ASVs – 29% for C4) whilst cluster 1 represented 9% (122 ASVs), cluster 2, 8% (116 ASVs), cluster 5, 17% (230 ASVs) and cluster 6 only 4% (48 ASVs) of the ASVs, respectively.

### Drivers of CFM abundance

**Fig. 6:**
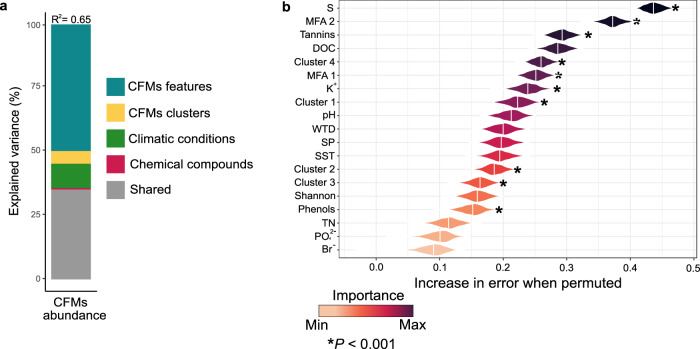
Drivers of absolute quantification of CO_2_-fixing microorganisms (CFMs) in peatlands. **a** Variation partitioning modeling evaluating the unique and shared portions of variation in CFM abundance. CFM features refer to ASV richness (S), alpha diversity (Shannon) and community composition (MFA axis 1 and 2); CFM clusters refers to relative abundance of species from clusters 1 to 4 generated by the JSDM model; Climatic conditions refer to WTD, SP and SST; Chemical compounds refer to tannins, phenols, DOC, K^+^, pH, TN, PO_4_^2-^ and Br^-^ and Shared refers to the percentage of shared variation explained by all predictors. **b** Results from random forest analysis showing the relative importance of the different drivers of the absolute quantification of CFMs in peatlands. WTD water table depth, SP spring precipitation, SST spring soil temperature, DOC dissolved organic carbon, TN total nitrogen.

Total CFM abundance was mainly driven by CFM features (diversity and community structure; 49% of variance explained; Fig. 6a), notably ASV richness (S) and community structure (MFA axis 1 and 2) (*P* < 0.001; Fig. 6b). However, an important part of total CFM abundance was shared by all drivers (35%; Fig. 6a). In particular, the relative abundance of species from clusters 1 and 4 together with tannins were important drivers (*P* < 0.001; Fig. 6b). CFM clusters, climatic conditions and chemical compound were not explaining an important part of the variance (5%, 9% and 2% of variance explained, respectively; Fig. 6a). Taken individually, we found that the abundance of each CFM pathway was driven by the same variables (Supplementary Figs. 15–17). Oxygenic phototrophic abundance was mostly driven by CFM features (46% of variance explained; ASV richness, MFA1 and MFA2), but also shared some variance with other drivers such as clusters 1, 2, 3, and 4 and tannins (49%, Supplementary Figs. 7g and 15). Chemoautotrophic abundance was explained by CFM features (44%), together with CFM clusters relative abundance (38%) and climatic conditions (18%) (Supplementary Fig. 16a). Among these groups of variables, community structure (MFA axis 1 and 2), alpha diversity (Shannon), observed richness (S), tannins and relative abundance of cluster 3 were again the most important drivers (*P* < 0.001; Supplementary Figs. 7g and 16b). Finally, AAnPB abundance was mostly related to CFM features (54% of variance explained; Supplementary Fig. 17a), with observed richness (S), alpha diversity (Shannon) and community structure (MFA axis 1 and 2) being the main drivers (*P* < 0.001; Supplementary Figs. 7g and 17b).

## Discussion

Our aim was to explore CFM abundance, diversity, and community composition as well as to identify their main environmental drivers. We show that CFMs constitute ~40% of the total bacterial abundance (Fig. 2a) regardless of the depth or the peatland type, which corroborates recent observations^20^. These findings highlight the potential of CFMs for carbon fixation in peatlands through the CBB cycle. The high abundance of CFMs was supported by a strong diversity spanning 37 phyla from 7960 ASVs. However, this diversity was not consistent among all sites. We found that CFMs were structured into different communities that were either present in a few sites or widespread across all sites according to environmental conditions. This emphasized the presence of a core microbiome that was modulating CFM diversity and abundances across sites. We caution that the high diversity of CFMs we are detecting does not guarantee CO_2_ fixation, as bacteria can be metabolically flexible with the ability to use various energy and carbon sources^25^. Whether this potential of fixation can be translated into a high CO_2_-fixation rate would require direct CO_2_ fixation measurements using, for example, isotopic labeling^26^. Nevertheless, this work is the first demonstration of the diversity and abundance of different CFM communities in peatlands, which lays the basis to better understand the role of microorganisms in peatland primary productivity and how they could contribute to peatland C sequestration^27^.

Our findings reveal that CFM abundance is very similar to that of other ecosystems despite very contrasting environmental conditions. Indeed, both oxygenic phototrophs and chemoautotrophs abundances were comparable to those found in deserts, forests, grasslands, or wetland soils, with around 10^5^–10^8^ copies.g^−1^DW (refs. ^28, 29^). AAnPB abundance was also similar to those found in soils (10^5^–10^9^ copies.g^−1^DW) (ref. ^30^). However, AAnPBs were found to be more abundant in peatlands than in aquatic systems, where previous studies had shown their low abundances ranging from 10^2^ to 10^5^ cells.mL^−1^ (ref. ^31^). We further expected depth to have an important impact on these abundances. Oxygenic phototrophs and AAnPBs are sensitive to the presence of light and oxygen, which are mostly available in the first top centimeters of peatland soil^24,32^, while chemoautotrophs are linked to the presence of reduced compounds more concentrated in deeper layers^2,24^. While we did observe a decrease in phototrophs with depth, we did not see a significant increase in chemoautotrophs. This shows that chemoautotrophs occur in surface peat and are not structured by depth in the first 20 cm of peat.

The high abundance of chemoautotrophs across the first 20 cm of peat suggests that they fix CO_2_, as microbial abundance is often assumed to be related to metabolic activity^21,33,34^. In particular, Huang et al.^26^ and Bay et al.^15^ showed that genes related to C fixation are largely transcribed by carbon fixing microorganisms in soils and that there is a link between the abundance of genes involved in CO_2_ fixation and CO_2_ fixation itself. AAnPBs were also abundant and as they have the potential to fix CO_2_^17^, they are likely significant player for the peatland C cycle. Thus, we speculate that chemoautotrophs and AAnPBs need to be considered in peatland primary productivity and that previous estimates, only based on oxygenic phototrophs^21^, most probably underestimate C fixation conducted by microorganisms. Moreover, it is important to note that our focus was solely on the CBB cycle, whereas CFMs utilize other pathways. For instance they often perform the Wood–Ljungdahl pathway that can be energetically favorable^10^, and which further increases the C fixation potential of CFMs.

We found that peatlands were characterized by a high CFM diversity. In particular, CFM classes that are commonly found in other soils^28,35^, such as Cyanophyceae, Palmophyllophyceae, Bacillariophyceae, Synurophyceae, Trebouxiophyceae (oxygenic phototrophs) and Nitrobacteraceae, Thiobacillaceae and Acidiferrobacteraceae (chemoautotrophs), were also predominant in peatlands. For AAnPBs we found one phylum, Vulcanimicrobiota also known as “Candidatus Eremiobacterota”, completely dominating the samples. This phylum has also been found in permafrost soils^36^, boreal mosses^37,38^ and other peatlands^39^. Bacteria of the phylum, Vulcanimicrobiota are metabolically diverse and versatile, performing photoautotrophy^37,38^ and chemolitoautotrophy^40^ allowing them to adapt to acidic and nutrient-poor environments^37,40^. Recently, Yabe et al.^17^ succeeded for the first time in the isolation of a “Candidatus Eremiobacterota” representative, *Vulcanimicrobium alpinus*. This species demonstrated bacteriochlorophyll biosynthesis, CO_2_ fixation and phototrophic motility capabilities. Thus, the presence of Vulcanimicrobia suggests that AAnPBs can fix CO_2_ in peatlands. As shown by Yabe et al.^17^, these AAnPBs may not rely on CO_2_ fixation for the CBB cycle; instead, they might utilize anaplerotic pathways to replenish the citric-acid cycle to maintain optimal functioning under nutrient poor conditions.

We found that CFM diversity can be structured into six main clusters, all shaped by specific, and sometimes antagonistic, responses to environmental conditions (Fig. 5). These clusters showed different ecological niche sizes (Fig. 5c) meaning that some clusters were ubiquitous (C3 and C4) while others were only present in a few sites (C1, C2, C5 and C6). Taken together, these findings demonstrated a core (C3 and C4) and a more specific CFM microbiome (C1, C2, C5, and C6). The core microbiome was strongly related to temperature, water table depth, pH, C and P. It was composed of a mix of families belonging to the three CFMs groups. The most abundant species retrieved were *Nitrobacter winogradsky* and *N. vulgaris*, which are important players of the C and N cycles^41^, *Anabaena sp*. a common carbon and nitrogen fixing cyanobacteria^42^, *Prasinoderma coloniale*, a green algae adapted to low light and oligotrophic habitats^43^ and Vulcanimicrobiia (AAnPBs). Species able to utilize H_2_ oxidation to derive energy^44^ like *Hydrogenophaga sp*. and *Cupriavidus metallireducens*, and species able to oxidize thiosulfate, tetrathionate as well as elemental sulfur^45^ such as *Sulfurisistis variabilis* and *Sulfuricaulis limicolica* were also found in this core microbiome. The specific microbiome greatly differs from the core microbiome, each cluster harboring different species and responding differently to local environmental conditions such as temperature, water table depth and nutrient availability. ASVs composing the specific CFM microbiome essentially belonged to Burkholderiaceae, Nitrobacteraceae and Thiobacillaceae families with two species more abundant, *Thiomonas sp*., adapted to low pH and low concentration of nutrients^46^ and *Thiobaccillus sp*., that fix CO_2_ through the oxidation of inorganic sulfur compounds^47^. Some very specific species were also retrieved such as *Acidihalobacter prosperus*, a chemoautotroph bacteria acidophile and halotolerant able to use either ferrous iron or reduced sulfur as electron donor^48^ to fix CO_2_ and *Chaetosphaeridium globosum*, a freshwater green algae^49^ that were only present in cluster 5.

Our results further reveal complex interactions between CFM abundances, richness, community structure and ASVs’ responses to environmental parameters. While CFM total abundance was directly driven by CFM diversity and community structure (Fig. 6), we found that environmental variables had rather indirect effects by modulating CFM community structure (Fig. 5). Indeed, oxygenic phototrophs, chemoautotrophs, and AAnPBs abundances were mainly driven by ASV richness, clusters relative abundance, and community structure. In turn, these CFM features were driven by environmental parameters. In particular, we found that climate (soil temperature and water content) was the main driver of CFM community structure. However, pH and nutrients such as DOC and TN were also important parameters (Fig. 5). Although only a few studies have assessed the drivers of CFM communities in soils, they identified similar drivers such as soil moisture, SOC, pH and temperature for oxygenic phototrophs^28^, and nutrients, TN, SOC, and climate for chemoautotrophs and CFMs globally^50,51^, thus corroborating our findings.

To sum up, carbon fixation in ecosystems is underpinned by at least seven biochemical pathways^10^. This study provides a comprehensive picture on how three major microbial groups able to perform carbon fixation are distributed among peatlands. It also demonstrates the unexpected abundance and occurrence of certain taxonomic groups. In particul ar, we evidenced the importance of the core CFM microbiome in modulating CFM diversity and abundance across environmental gradients. We caution that our study is DNA-based and therefore does not demonstrate activity. As such, further studies are needed to relate CFM community and diversity to actual CO_2_ fixation rates^52^. Nevertheless, our findings have important implications for peatlands, as they show that a high proportion of bacteria and archaea have the potential to contribute to peatland carbon fixation than previously thought. They also show that environmental changes can restructure these communities, and in particular hydrological and temperature changes, and thus modulate CFM abundances. Climate change could drive important CFM-feedbacks for the peatland C cycle. To illustrate, Hamard et al.^9^ recently showed that oxygenic photoautotrophic CO_2_ fixation strongly increased with warming, thereby mitigating C emissions. This suggests that, overall, CFMs could potentially mitigate predicted peatland C emissions to a greater extent^8,9^, highlighting the importance of considering not only oxygenic phototrophs but also chemoautotrophs and AAnPBs in peatland C fixation.

## Methods

### Study sites and sampling

We selected four peatlands along a latitudinal gradient spanning different environmental conditions and trophic states, from northern Sweden to southern France. Counozouls (42°41’16N - 2°14’18E; 1350 m above sea level (a.s.l)) is a moderately rich fen in the Pyrenees mountains, southwestern France. This site is characterized by a mean annual temperature (MAT) and annual precipitation (MAP) of 7.1 °C and 1027 mm, respectively. Männikjärve (58°52’30N - 26°15’04E; 82 m a.s.l) is an ombrotrophic bog situated in Central Estonia in the Endla mire system and characterized by MAT of 5.9 °C and MAP of 623 mm. Siikaneva (61°50’00N - 24°11’21E; 160 m a.s.l) is a boreal oligotrophic fen in southern Finland with MAT and MAP of 5.1 °C and 611 mm, respectively. Abisko (68°20’54N - 19°04’09E; 350 m a.s.l) is a palsa mire characterized by MAT of 2.7 °C and MAP of 418 mm. More details on these sites can be found in Sytiuk et al.^53^.

At each site, we collected five peat cores (10 cm diameter, 20 cm depth) in homogeneous *Sphagnum* habitats during summer 2022. Peat cores were further subdivided into three depths (undecomposed *Sphagnum* layer, D1: 0–5 cm; poorly decomposed *Sphagnum* layer, D2: 5–10 cm; and moderately decomposed *Sphagnum* layer, D3: 10–15 cm), representing layers with increasing amount of organic matter decomposition with increasing depth. In total we had 60 samples, 15 for each site with 5 per depth. At each depth, a few grams of peat were collected, cut into small pieces, homogenized, and placed in sterile 5 mL Eppendorf tubes containing 3 mL of RNA*later* (ThermoFisher) for environmental DNA analysis. The remaining peat was placed in sterile plastic bags for chemical analyses. We also sampled pore water to complete the chemical characterization. All samples were stored at −20 °C prior proceeding to DNA extraction and chemical analyses.

### Collection of environmental data

Daily air and soil temperature, precipitation and water table depth were measured at hourly intervals at each site since 2019 (Meters® sensors and data loggers; Meter Group, Pullman, WA, USA). Average air temperature, soil temperature, precipitation and water table depth for the spring period (20^th^ of March – 21^st^ of June) and for the winter period (21^th^ of December – 19^th^ of March) were calculated using these data. Pore water was collected in piezometers at each site and filtered through 0.45 µm pore size (Whatman) to measure pH, dissolved organic carbon (DOC), total nitrogen (TN) and the quality of the dissolved organic matter. DOC and TN were measured by combustion on a Shimadzu TOC-L. Dissolved organic matter quality was assessed by measuring the aromatic content and molecular weight of dissolved organic carbon. To do so, we measured the absorbance of DOC between 250 and 660 nm (15 wavelengths in total) in 200 µL sample aliquots in 96-well quartz microplate using a BioTek SynergyMX spectrofluorometer^54^. We retrieved relative fluorescence efficiency (RFE), freshness, fluorescence index (FI), aromatic peaks and biological index (BIX) to assess the quality of the organic matter. Demineralized water filtered through Whatman filter was used as a blank and to correct the values. Peat soil water content was measured by weighing peat samples fresh and dry (lyophilized samples; expressed per g of H_2_O per g of dry peat (gH_2_O.g.^−1^ DW)). Peat samples were further used to measure several cations (Li^+^, Na^+^, NH_4_^+^, K^+^, Mg^2+^ and Ca^2+^) and anions (F^-^, Cl^-^, NO_2_^-^, NO_3_^-^, Br^-^, SO_4_^2-^ and PO_4_^3-^) using high performance ion chromatography on a Dionex DX-120 and on a Dionex ICS-5000+ respectively. Finally, we quantified total concentrations of carbohydrates, flavonoids, tannins, phenols and water phenols in the peat following Sytiuk et al.^53^.

### DNA extraction, amplification and sequencing

DNA was extracted using the DNeasy PowerSoil Pro Kit (Qiagen) following manufacturer’s instructions. DNA concentration was quantified using a Nanodrop ND-1000 spectrophotometer (Thermo Fisher Scientific^TM^). Extracts were then stored at −20 °C prior to DNA amplification. For all samples we targeted four different genes, namely the 16S rRNA gene, the 23S rRNA gene, *cbbL* and *bchY*. The 16S rRNA gene allowed to investigate all prokaryotes while the others genes were used to target CFMs using the CBB cycle: the 23S rRNA gene for microorganisms involved in oxygenic photosynthesis, *cbbL* gene encoding the large subunit of RuBisCO form IA for chemoautotrophs^55,56^ and *bchY* encoding the Y subunit of bacteriochlorophyll biosynthesis for AAnPBs^57^. The primers pairs used for DNA amplification (Supplementary Table 8a) were PCR1-515F/PCR1-909R for the 16S rDNA gene, P23SrV-f1/P23Srv-1 for the 23S rDNA gene, cbbL-IA-CHEM/cbbL-IA-r for *cbbL* and bchY-fwd/bchY-rev to target *bchY*. All primers contained additional Illumina adapters and tags. PCRs were performed in a total volume of 50 μL containing 25 μL of AmpliTaq Gold^TM^ Master Mix (applied biosystem, ThermoFisher), 21 or 19 μL of ultrapure water, 1 μL of forward and 1 μL of reverse primer (final concentration of 20 μM) and 2 or 4 μL of DNA template according to initial DNA concentration. The PCR reaction conditions were different according to each primer pair (Supplementary Table 9). PCR quality was assessed using 1.65% agarose gel electrophoresis. The high throughput sequencing was performed by the GeT-PlaGe platform (Genotoul, Toulouse, France) using Illumina MiSeq technology with the V3 chemistry. To avoid contamination as much as possible, all molecular work was carried out in a dedicated laboratory using UV-decontaminated and sterile materials. Blanks were also included throughout the process to check for potential contamination.

### Analysis of CFM communities

Sequences obtained after sequencing were already demultiplexed, trimmed of barcodes and Illumina adapters. The paired-end fastq sequences were analyzed within the FROGs pipeline v4.1.0 provided by the Galaxy web platform^58^. Paired-end sequences were merged using *VSEARCH* v2.17.2^59^. Sequences were then denoised, dereplicated, clustered into ASVs using SWARM clustering method^60^ and chimera were removed. These ASVs were further filtered to keep only ASVs with a minimum prevalence of 2 and assigned at different taxonomic levels using the database *SILVA 138.1*^61^ for the 16S rRNA gene and *µgreen db algae v1.2*^62^ for the 23S rRNA gene. For *cbbL* and *bchY* no databases were available, and therefore affiliations were done manually. First, sequences were aligned using Clustal OMEGA (EMBL), clustered using the *hclust* function (threshold = 0.05) and for each cluster a consensus sequence was built. Each consensus sequence was then blasted using nucleotide BLAST for highly similar sequences (megablast). Only assignations with high score, query cover (> 90%), percentage identity (> 90%) and low E-value (< 0.01), were kept while other sequences were considered as “*unassigned*”. All the ASVs obtained were rarefied on the basis of the total number of reads for the sample with the lowest number of reads using the *rarefy_even_depth* function of the *Phyloseq* R package v1.44.0 to obtain comparable sequencing depth across samples (Supplementary Fig. 18).

### Absolute quantification using digital droplet PCR (ddPCR)

Absolute quantification of genes targeting prokaryotes, oxygenic phototrophs, chemoautotrophs and AAnPBs was measured using digital droplet PCR (ddPCR, BioRad) as described in Le Geay et al.^20^. Primers pairs used for ddPCR (Supplementary Table 8b) were L/Prba338f with K/Prun518r for prokaryotes, 16SCF/16SUR for cyanobacteria, 23S255f/ P23SrV_r1 for green algae and cyanobacteria, cbbLR1F/cbbLR1inR for chemoautotrophs, and pufMforward557/pufMreverse750 for AAnPBs. We did not use *bchY* primers to quantify AAnPBs because they were too long and too degenerate to be used in ddPCR^57,63^. A DX200 instrument (BioRad) was used to run the ddPCR reactions in a total volume of 20 µL with 10 µL of EvaGreen Supermix (BioRad, 1X), 0.5 µL of each primer (final concentration 25 µM) and 4 µL of ultrapure water. Template DNA was diluted either at 1/10, 1/100 or 1/1000 and 5 µL were added to the reactional mix. We then used the QX200 Droplet Generator (BioRad) with QX200 Droplet Generation Oil for EvaGreen (BioRad) to emulsify the reaction mix and transferred the mix into a 96-well PCR plate. The plate was heat-sealed with a foil seal and then placed on a C1000 Touch Thermocycler with deep-well module (BioRad) to run the PCR (detailed PCR programs are described in Supplementary Table 10). Following amplification, plates were equilibrated for at least 10 min at room temperature. Then, the fluorescence was read on a QX200 Droplet Reader (BioRad) and the QuantaSoft software was used to set the threshold and analyze the results. Using ultrapure water as a negative control and different DNA extracted from cultures of microorganisms as positive controls we manually defined the threshold for each ddPCR run. *Escherichia coli* and *Micromonas pusilla* DNA diluted 1/100 were used as positive controls for prokaryotes and oxygenic phototrophs respectively. Calculation of the final concentration considered the volume of eluted DNA (70 µL), the volume of ddPCR reaction mixture (20 µL), the volume of template DNA (5 µL) and the dilution factor of the template DNA (10, 100 or 1000). Results were further normalized using the amount of dry peat used for DNA extraction to obtain a final concentration in target copies.g^−1^ of dry peat (copies.g^−1^ DW).

### Statistical analysis

All statistical analyses were performed using Rstudio v12.0 with R build under v4.3.2 with packages specified below and graphical representations done using *ggplot2* v3.5.1 and *igraph* v1.4.3 (see Supplementary Table 11 for details about packages and functions). When applicable, statistical significance was added directly on graphs using the function *stat_compare_means* of the package *ggpubr* v0.6.0.

Environmental drivers of samples distribution were tested by conducting a principal component analysis (PCA) using the *PCA* function of *FactoMineR* package v2.11 including all the environmental parameters. Environmental parameters were logged (log10) or squared-root transformed to ensure normality. For further analyses, environmental parameters went through a selection of variables to retain only the most significant and least collinear representative for climatic variables, nutrients, metabolites and organic matter quality variables. Collinearity was further assessed using *corrplot* of the *corrplot v0.92* package (Supplementary Fig. 19). Correlation between absolute quantification of oxygenic phototrophs, chemoautotrophs and AAnPBs genes and environmental variables was assessed using *corrplot* of the *corrplot v0.92* package with Pearson correlation coefficient.

For each CFM marker gene (23S rRNA, *cbbL* and *bchY* genes), richness and alpha-diversity were estimated using ASVs observed richness and Shannon metric (*vegan* v2.6.4), respectively. Linear mixed effect models (LME) using the *lme* function of the *nlme* package v3.1-164 were implemented to test the impact of peatland site and depth on these diversity metrics and on absolute gene quantification. To account for the repeated measures, we used plot nested in site as a random intercept effect. The normality and homoscedasticity of linear mixed effects models residuals were visually assessed. To estimate species turnover between sites and depths, we also conducted a non-metric multidimensional scaling (NMDS) based on a beta-diversity metric (Bray-Curtis dissimilatory) using the function *metaMDS* of the vegan package v2.6.4. We further used permutational multivariate analysis of variance (PERMANOVA) to test for structural difference in the communities according to location and depth using the function *adonis2* of the *vegan* package v2.6-4. Correlation between selected environmental variables and diversity metrics was assessed using *corrplot* with Pearson correlation coefficient.

Multiple factor analysis (MFA) was used to test the general structure of the three CFM communities, *i.e*., oxygenic phototrophs, chemoautotrophs and AAnPBs. MFA allows the coupling of several species matrices^64^ (oxygenic phototroph ASVs, chemoautotroph ASVs and AAnPB ASVs). To do so, the ASV matrix of each gene was first transformed using a Hellinger transformation, and then, a MFA was computed on this assembled species matrix using the *MFA* function of the package *FactoMineR* v2.11. Correlations between the three species matrices were measured using the RV coefficients (Pearson’s correlation coefficient). Euclidean distances of the MFA site scores were further used to perform cluster analysis using *hclust* with the Ward D2 method. The resulting dendrogram was plotted in the MFA ordination space.

To test the impact of location and depth on the relative abundance of CFMs, ASVs were aggregated by taxonomic rank and the relative abundance of each group (Class and Family) was calculated. Based on these relative abundances at each location and for each depth, heatmaps were generated using *geom_tile* of the ggplot2 package. Linear mixed effect models (LME) using the *lme* function of the *nlme* package v3.1-164 were implemented with location and depth as fixed explanatory terms to explain the relative abundance of each group and replicates were used as a random term.

To further identify the patterns of occurrence among CFM ASVs and their joint responses to environmental conditions, we used a joint species distribution model (JSDM)^65^. JSDM uses a hierarchical approach to link species abundance with their similarity in response to environmental factors in order to identify species co-response to environmental conditions. The model is based on analysing the joint distribution of species and uses inverse predictions to measure the influence of environmental factors on multiple species simultaneously^66^. The identified species relationships can be further divided into those caused by a similar response to environmental factors and those unrelated to environmental factors (*i.e*. residual correlations). To perform the JSDM, we, first, standardized environmental parameters using the *decostand* function of the *vegan* package v2.6.4, and we filtered the ASVs to retain those with a minimum relative abundance of 0.1%. Next, we ran the JSDM model using the *JSDM_binomial_probit* function of the *jSDM* package v0.2.6 on presence/absence data^65^. The model was checked using the diagnostic plots provided by the package. We obtained the correlation matrix among ASVs, representing their joint response to environmental parameters, using *get_enviro_cor* function of the *jSDM* package v0.2.6. Using this correlation matrix, we then built the co-occurrence network using the function *cluster_fast_greedy* of the *igraph* package v1.4.3 based on the strongest correlation link. We also recovered the beta-coefficient of the JSDM model to show the specific response of each ASV to every environmental factor. For each cluster, we further analyzed the taxonomic affiliation of the ASVs belonging to the cluster. The relative abundances of the six main clusters generated by the JSDM package were recovered for each sample and projected into the multiple factor analysis (MFA) ordination space. The size of niche spaces of the ASVs in each identified JSDM cluster was finally estimated by calculating hypervolumes using *hypervolumes* R package v3.1.4^67^. We defined the n-dimensional hypervolume of each ASV using the site scores of the first three axes of the principal component analysis describing environmental conditions in each site (see Fig. 1).

To identify the main drivers of CFM abundances, we first used variation partitioning modeling. We quantified the relative importance of CFM features (richness, diversity, community structure), relative abundance of CFM clusters (C1, C2, C3 and C4 that were the most abundant), climatic (water table depth, spring precipitation and spring soil temperature) and chemical (phenols, tannins and several nutrients) factors in driving CFM abundances (total and of each specific gene). We used the *varpart* function from the *vegan* package v2.6-4. This function identifies the unique and shared portions of the variation in the distribution of the response variable explained by the four groups of factors mentioned above, while avoiding multicollinearity as it partitions the variance of the response variable attributed to a particular group of predictors. Secondly, we used random forest analysis^68^ to identify the variables of importance that modulate CFM abundances, total and of each specific gene. Random forest model was computed using the same variables than for variance partitioning. Random forest is an ensemble of learning method that builds multiple classification trees using binary splits^69^. Each tree is trained on a random subset of the data, typically using about two-thirds of the observations, while the remaining one-third—known as out-of-bag (OOB) samples—are withheld for validation. Predictor importance is assessed by measuring the increase in mean squared error between observed and OOB-predicted values when the values of a given predictor are randomly permuted. The overall importance of each predictor is calculated as the average decrease in prediction accuracy across all trees^69^. This analysis was performed using the function *plot_importance* of the *R* package *spatialRF* v1.1.4, which enhances model performance by reducing multicollinearity, identifying relevant variable interactions, and evaluating model transferability through spatial cross-validation.

## Supplementary information

Supplementary Information

## Data Availability

The datasets generated during and/or analysed during the current study are available in the following Zenodo repository: 10.5281/zenodo.15342859.

## Code availability

The code generated during the current study are available in https://github.com/vjassey/Diversity_CFMs_peatlands.
