# Supplementary material for: Uncovering diversity and abundance patterns of CO2-fixing microorganisms in peatlands
Source: NPJ Biodivers. 2025 Aug 4;4:30. doi: 10.1038/s44185-025-00099-1 (PMC12322008; doi:10.1038/s44185-025-00099-1)
Supplement: Supplementary file 1 — Supplementary Information [file 44185_2025_99_MOESM1_ESM.pdf]

Supplementary Information

Uncovering diversity and abundance patterns of CO_2-fixing microorganisms in peatlands

Marie Le Geay[1*], Kyle Mayers[2], Anna Sytiuk[1], Ellen Dorrepaal[3], Martin Küttim[4], Mariusz Lamentowicz[5], Eeva-Stiina Tuittila[6], Béatrice Lauga[7], and Vincent E.J. Jassey[1*]

[1] Université de Toulouse, Toulouse INP, CNRS, IRD, CRBE, Toulouse, France

[2] Molecular Ecology and Paleogenomics - MEP, NORCE, Bergen, Norway

[3] Climate Impacts Research Centre, Department of Ecology and Environmental Science, Umeå University, Abisko, Sweden

[4] Institute of Ecology, School of Natural Sciences and Health, Tallinn University, Uus-Sadama 5, Tallinn, Estonia

[5] Climate Change Ecology Research Unit, Adam Mickiewicz University, Poznań, Poland

[6] School of Forest Sciences, University of Eastern Finland, Joensuu, Finland

[7] Universite de Pau et des Pays de l'Adour, E2S UPPA, CNRS, IPREM, Pau, France

*corresponding authors: marie.le-geay@univ-tlse3.fr and vincent.jassey@univ-tlse3.fr

Supplementary Table. 1| Environmental and chemicals characterization of each site. Cou = Counozouls; Män = Männikjärve; Siik = Siikaneva; Abi = Abisko; DOC = dissolved organic carbon; TN = total nitrogen; WAT = winter air temperature; WST = winter soil temperature; WP = winter precipitation; WWTD = winter water table depth; SAT = spring air temperature; SST = spring soil temperature; SP = spring precipitation and SWTD = spring water table depth.

	pH	DOC	TN	WAT	WST	WP	WWTD	SAT	SST	SP	SWTD
**Cou**	5.8	83.8	1.8	-1.3	1.4	85.0	462.0	6.4	7.1	275.0	472.1
**Män**	5.0	44.4	1.3	-4.7	0.9	105.0	358.6	5.5	5.5	142.0	391.3
**Siik**	4.9	32.5	0.7	-6.0	0.2	0.0	681.2	2.5	3.1	177.0	768.2
**Abi**	4.7	52.3	1.4	-10.2	-0.6	5.0	40.8	2.9	2.0	26.0	114.5

Supplementary Table. 2| Results of linear mixed effect models (LME) testing the impact of location and depth on different environmental parameters including metabolites, organic matter quality, nutrients and climatic conditions. Cou = Counozouls, Män = Männikjärve, Siik = Siikaneva, Abi = Abisko, DOC = dissolved organic carbon, TN = total nitrogen, WAT = winter air temperature, WST = winter soil temperature, WP = winter precipitation, WWTD = winter water table depth, WPAR = winter PAR, SAT = spring air temperature, SST = spring soil temperature, SP = spring precipitation, SWTD = spring water table depth, SPAR = spring PAR, D1 = 0 - 5 cm, D2 = 5 - 10 cm, D3 = 10 - 15 cm. Significant *p-values* in bold.

	Location		Depth Cou		Depth Män		Depth Siik		Depth Abi	
	F-value	*p-value*	*F-value*	*p-value*	*F-value*	*p-value*	*F-value*	*p-value*	*F-value*	*p-value*
DOCq	0.95	0.45	-	-	-	-	-	-	-	-
Peak_A	4.93	**0.019**	-	-	-	-	-	-	-	-
Peak_C	4.54	**0.024**	-	-	-	-	-	-	-	-
Peak_M	4.12	**0.032**	-	-	-	-	-	-	-	-
RFE	5.36	**0.014**	-	-	-	-	-	-	-	-
Freshness	18.45	**<0.001**	-	-	-	-	-	-	-	-
BIX	11.43	**<0.001**	-	-	-	-	-	-	-	-
FI	3.09	0.068	-	-	-	-	-	-	-	-
WTD	72.53	**<0.001**	-	-	-	-	-	-	-	-
Tannins	2.72	0.091	5.61	**0.03**	15.61	**0.0017**	4.04	0.061	1.8	0.23
Water_Phenols	0.33	0.80	0.24	0.79	2.62	0.13	5.44	**0.032**	0.48	0.63
Carbohydrates	5.85	**0.011**	1.79	0.23	12.67	**0.0033**	0.5	0.63	2.46	0.15
Flavonoids	0.19	0.90	0.74	0.51	4.47	**0.05**	0.88	0.45	0.88	0.45
Phenols	1.5	0.26	2.81	0.12	8.29	**0.011**	1.86	0.22	0.28	0.76
Na^+	0.33	0.80	3.94	0.065	0.66	0.54	1.12	0.37	0.23	0.8
NH_4^+	1.75	0.21	1	0.41	0.68	0.53	1.07	0.39	0.37	0.70
K^+	1.67	0.23	9.22	**0.0084**	13.63	**0.0026**	31.56	**<0.001**	8.14	**0.012**
Mg^{2+}	13.31	**<0.001**	0.5	0.62	1.13	0.37	0.044	0.96	2	0.2
Ca^{2+}	1.51	0.26	1.3	0.32	1.78	0.23	0.8	0.48	0.90	0.44
F^-	2.86	0.081	6.23	**0.023**	0.57	0.59	0.88	0.45	0.97	0.42
Cl^-	0.7	0.57	2	0.2	0.89	0.45	0.44	0.66	0.69	0.53
NO_2^-	0.38	0.77	2.24	0.17	1	0.38	3.12	0.099	0.54	0.6
NO_3^-	1.84	0.19	1.52	0.28	7.19	**0.016**	1.62	0.26	0.58	0.58
Br^-	4.19	0.03	4.33	0.053	3.36	0.087	3.5	0.081	11.81	**0.0041**
SO_4^{2-}	0.75	0.54	0.17	0.85	0.55	0.6	1.26	0.33	0.17	0.85
PO_4^{3-}	25.18	**<0.001**	2.13	0.18	1.47	0.29	1.11	0.37	1.61	0.26
pH	7.09	**0.0054**	-	-	-	-	-	-	-	-

DOC	10.38	**0.0012**	-	-	-	-	-	-	-	-
TN	7.61	**0.0041**	-	-	-	-	-	-	-	-
WAT	>100	**<0.001**	-	-	-	-	-	-	-	-
WP	>100	**<0.001**	-	-	-	-	-	-	-	-
WST	>100	**<0.001**	-	-	-	-	-	-	-	-
WPAR	>100	**<0.001**	-	-	-	-	-	-	-	-
WWTD	>100	**<0.001**	-	-	-	-	-	-	-	-
SAT	>100	**<0.001**	-	-	-	-	-	-	-	-
SP	>100	**<0.001**	-	-	-	-	-	-	-	-
SST	>100	**<0.001**	-	-	-	-	-	-	-	-
SPAR	>100	**<0.001**	-	-	-	-	-	-	-	-
SWTD	>100	**<0.001**								

Supplementary Table. 3| Results of posthoc test (after LME) testing the impact of location and depth on different environmental parameters including metabolites, organic matter quality, nutrients and climatic conditions. Cou = Counozouls, Man = Männikjärve, Siik = Siikaneva. Abi = Abisko, DOC = dissolved organic carbon, TN = total nitrogen, WAT = winter air temperature, WST = winter soil temperature, WP = winter precipitation, WWTD = winter water table depth, WPAR = winter PAR, SAT = spring air temperature, SST = spring soil temperature, SP = spring precipitation, SWTD = spring water table depth, SPAR = spring PAR, Cou = Counozouls, Män = Männikjärve, Siik = Siikaneva. Abi = Abisko, D1 = 0 - 5 cm, D2 = 5 - 10 cm, D3 = 10 - 15 cm. Significant *p-values* in bold.

	Location						Depth Cou			Depth Män			Depth Siik			Depth Abi		
	Cou-Män	Cou-Siik	Cou-Abi	Män-Siik	Män-Abi	Abi-Siik	**D1-D2**	**D1-D3**	**D2-D3**	**D1-D2**	**D1-D3**	**D2-D3**	**D1-D2**	**D1-D3**	**D2-D3**	**D1-D2**	**D1-D3**	**D2-D3**
DOCq	0.73	0.66	0.4	0.99	0.93	0.96	-	-	-	-	-	-	-	-	-	-	-	-
Peak_A	0.97	0.97	**0.043**	0.82	0.09	**0.02**	-	-	-	-	-	-	-	-	-	-	-	-
Peak_C	0.89	0.93	0.062	0.57	0.2	**0.02**	-	-	-	-	-	-	-	-	-	-	-	-
Peak_M	0.99	0.56	0.21	0.73	0.14	**0.023**	-	-	-	-	-	-	-	-	-	-	-	-
RFE	0.98	**0.026**	0.094	0.053	0.18	0.87	-	-	-	-	-	-	-	-	-	-	-	-
Freshness	**0.0004**	**0.0001**	**0.0012**	0.73	0.92	0.38	-	-	-	-	-	-	-	-	-	-	-	-
BIX	**0.0076**	**0.0005**	**0.017**	0.40	0.96	0.21	-	-	-	-	-	-	-	-	-	-	-	-
FI	0.82	0.13	0.097	0.45	0.36	0.99	-	-	-	-	-	-	-	-	-	-	-	-
WTD	**<0.001**	**<0.001**	**<0.001**	0.99	0.92	0.84	-	-	-	-	-	-	-	-	-	-	-	-
Tannins	0.15	0.13	0.84	0.99	0.48	0.44	0.28	**0.02**	0.27	**0.021**	**0.0014**	0.16	0.26	0.053	0.54	0.21	0.45	0.84
Water_Phenols	0.94	1	0.96	0.94	0.76	0.97	0.78	0.96	0.91	0.99	0.17	0.18	**0.034**	0.084	0.82	0.76	0.98	0.64
Carbohydrates	0.31	**0.0097**	0.91	0.21	0.67	**0.032**	0.28	0.29	0.99	**0.026**	**0.0029**	0.28	0.93	0.6	0.81	0.29	0.15	0.88
Flavonoids	1	0.96	0.94	0.96	0.94	0.99	0.83	0.48	0.81	0.087	0.064	0.98	0.5	0.53	0.99	0.49	0.99	0.55
Phenols	0.72	0.78	0.93	0.24	0.96	0.44	0.35	0.11	0.67	**0.037**	**0.012**	0.73	0.27	0.24	1	0.86	0.98	0.75
Na^+	0.99	0.93	0.99	0.86	0.99	0.8	0.1	0.086	0.99	0.56	0.65	0.99	0.39	0.98	0.5	0.99	0.87	0.8
NH_4^+	0.24	0.99	0.99	0.37	0.29	0.99	1	0.47	0.47	0.5	0.81	0.85	0.99	0.44	0.46	0.91	0.68	0.9
K^+	0.99	0.34	0.99	0.43	0.97	0.23	**0.02**	**0.011**	0.90	**0.016**	**0.0025**	0.39	**0.0005**	**0.0002**	0.79	**0.046**	**0.012**	0.62
Mg^{2+}	**0.0015**	**0.032**	0.98	0.34	**0.0009**	**0.017**	0.66	0.99	0.69	0.34	0.74	0.74	0.99	0.97	0.96	0.63	0.57	0.17
Ca^{2+}	0.83	0.45	0.97	0.90	0.6	0.26	0.29	0.71	0.7	0.3	0.27	0.99	0.99	0.81	0.58	0.78	0.79	0.41
F^-	0.46	0.21	0.97	0.94	0.26	0.11	**0.026**	0.059	0.85	0.93	0.78	0.57	0.49	0.99	0.54	0.44	0.98	0.54
Cl^-	0.85	0.97	0.88	0.63	0.99	0.67	0.19	0.84	0.39	0.48	0.55	0.99	0.99	0.71	0.7	0.55	0.64	0.99
NO_2^-	0.97	1	0.81	0.97	0.96	0.79	0.21	0.24	0.99	0.74	0.36	0.76	0.99	0.14	0.13	0.63	0.99	0.69

NO_3^-	0.98	0.99	0.23	0.99	0.37	0.26	0.29	0.95	0.42	0.95	**0.034**	**0.022**	0.694	0.26	0.4	0.56	0.78	0.93
Br^-	0.48	1	**0.045**	0.48	0.43	**0.045**	0.16	**0.05**	0.7	0.56	0.076	0.33	0.87	0.085	0.17	0.97	**0.0087**	**0.0063**
SO_4^{2-}	0.80	0.99	0.57	0.89	0.98	0.68	0.98	0.84	0.92	0.71	0.98	0.60	0.53	0.33	0.91	0.91	0.99	0.84
PO_4^{3-}	**0.01**	**0.019**	**0.014**	0.98	**<0.0001**	**0.0001**	0.18	0.35	0.87	0.41	0.3	0.97	0.99	0.4	0.48	0.36	0.28	0.98
pH	**0.046**	**0.024**	**0.0044**	0.98	0.55	0.76	-	-	-	-	-	-	-	-	-	-	-	-
DOC	**0.007**	**0.0009**	**0.03**	0.62	0.84	0.22	-	-	-	-	-	-	-	-	-	-	-	-
TN	0.29	**0.0027**	0.53	0.069	0.96	**0.03**	-	-	-	-	-	-	-	-	-	-	-	-
WAT	**<0.0001**	**<0.0001**	**<0.0001**	**<0.0001**	**<0.0001**	**<0.0001**	-	-	-	-	-	-	-	-	-	-	-	-
WP	**<0.0001**	**<0.0001**	**<0.0001**	**<0.0001**	**<0.0001**	**<0.0001**	-	-	-	-	-	-	-	-	-	-	-	-
WST	**<0.0001**	**<0.0001**	**<0.0001**	**<0.0001**	**<0.0001**	**<0.0001**	-	-	-	-	-	-	-	-	-	-	-	-
WPAR	**<0.0001**	**<0.0001**	**<0.0001**	**<0.0001**	**<0.0001**	0.098	-	-	-	-	-	-	-	-	-	-	-	-
WWTD	**<0.0001**	**<0.0001**	**<0.0001**	**<0.0001**	**<0.0001**	**<0.0001**	-	-	-	-	-	-	-	-	-	-	-	-
SAT	**<0.0001**	**<0.0001**	**<0.0001**	**<0.0001**	**<0.0001**	**<0.0001**	-	-	-	-	-	-	-	-	-	-	-	-
SP	**<0.0001**	**<0.0001**	**<0.0001**	**<0.0001**	**<0.0001**	**<0.0001**	-	-	-	-	-	-	-	-	-	-	-	-
SST	**<0.0001**	**<0.0001**	**<0.0001**	**<0.0001**	**<0.0001**	**<0.0001**	-	-	-	-	-	-	-	-	-	-	-	-
SPAR	**<0.0001**	**<0.0001**	**<0.0001**	**<0.0001**	**<0.0001**	**<0.0001**	-	-	-	-	-	-	-	-	-	-	-	-
SWTD	**<0.0001**	**<0.0001**	**<0.0001**	**<0.0001**	**<0.0001**	**<0.0001**	-	-	-	-	-	-	-	-	-	-	-	-

Supplementary Table. 4| Results of linear mixed effect models (LME) testing the impact of location and depth on gene abundance. Cou = Counozouls, Männ = Männikjärve, Siik = Siikaneva. Abi = Abisko. Significant *p-values* in bold.

	Location		Depth Cou		Depth Män		Depth Siik		Depth Abi	
	F-value	*p-value*	*F-value*	*p-value*	*F-value*	*p-value*	*F-value*	*p-value*	*F-value*	*p-value*
16S rRNA	11.93	**<0.001**	22.16	**<0.001**	2.104	0.18	0.3	0.75	0.959	0.42
23S rRNA	3.489	**0.05**	4.47	**0.05**	75.52	**<0.001**	8.75	**0.008**	23.12	**<0.001**
cbbL	9.036	**0.0021**	12.66	**0.003**	31.15	**<0.001**	4.18	0.06	1.19	0.35
pufM	4.973	**0.018**	3.81	0.069	36.92	**<0.001**	2.77	0.12	8.3	**0.011**
16S rRNA (cyanobacteria)	4.629	**0.023**	6.42	**0.022**	173.86	**<0.001**	6.6	**0.02**	20.9	**<0.001**

Supplementary Table. 5| Results of posthoc test (after LME) testing the impact of location and depth on gene abundance. Cou = Counozouls, Männ = Männikjärve, Siik = Siikaneva. Abi = Abisko, D1 = 0 - 5 cm, D2 = 5 - 10 cm, D3 = 10 - 15 cm. Significant *p-values* in bold.

	Location						Depth Cou			Depth Män			Depth Siik			Depth Abi		
	Abi - Cou	Abi - Män	Abi - Siik	Cou - Män	Cou - Siik	Män - Siik	**D1 - D2**	**D1 - D3**	**D2 - D3**	**D1 - D2**	**D1 - D3**	**D2 - D3**	**D1 - D2**	**D1 - D3**	**D2 - D3**	**D1 - D2**	**D1 - D3**	**D2 - D3**
16S RNA	**0.041**	0.16	0.28	**<0.001**	**0.002**	0.98	**0.043**	**<0.001**	**0.015**	0.94	0.2	0.3	0.88	0.96	0.73	0.39	0.8	0.74
23S RNA	0.15	0.86	0.91	**0.04**	0.38	0.5	0.19	0.61	**0.045**	**<0.001**	**<0.001**	**<0.001**	0.56	**0.009**	**0.042**	**0.004**	**<0.001**	0.2
cbbL	0.16	0.061	0.46	**0.002**	**0.012**	0.68	**0.043**	**0.003**	0.16	0.71	**<0.001**	**<0.001**	0.16	0.76	0.056	0.43	0.4	0.99
pufM	0.11	0.63	0.99	**0.013**	0.088	0.7	0.37	0.058	0.42	**0.043**	**<0.001**	**0.002**	0.94	0.22	0.13	0.13	**0.01**	0.21
16S rRNA (cyanobacteria)	**0.05**	0.97	0.73	**0.023**	0.27	0.47	**0.021**	0.65	0.079	**<0.001**	**<0.001**	**<0.001**	0.78	**0.002**	0.058	**<0.001**	**<0.001**	0.99

Supplementary Table. 6| Results of linear mixed effect models (LME) testing the impact of location and depth on 23S rRNA gene, *cbbL* gene and *bchY* gene observed richness (S) and alpha diversity (Shannon index). Cou = Counozouls, Männ = Männikjärve, Siik = Siikaneva. Abi = Abisko. Significant *p-values* in bold.

		Location		Depth Cou		Depth Män		Depth Siik		Depth Abi	
		F-value	*p-value*	*F-value*	*p-value*	*F-value*	*p-value*	*F-value*	*p-value*	*F-value*	*p-value*
23S rRNA gene (oxygenic phototrophs)	S	6.19	**0.009**	7.6	**0.014**	30.92	**<0.001**	1.59	0.26	0.36	0.71
	Shannon	1.84	0.19	7.91	**0.013**	15.83	**0.002**	1.9	0.22	1.04	0.4
cbbL gene (chemoautotrophs)	S	11.57	**<0.001**	11.46	**0.005**	144.2	**<0.001**	4.54	**0.048**	54.4	**<0.001**
	Shannon	4.07	**0.033**	8.34	**0.011**	66.79	**<0.001**	2.96	0.11	29.55	**<0.001**
bchY gene (AAnPBs)	S	2.6	0.1	1.4	0.3	35.11	**<0.001**	2.97	0.11	5.31	**0.034**
	Shannon	9.77	**0.002**	4.06	0.061	8.04	**0.012**	15.55	**0.002**	2.68	0.13

Supplementary Table. 7| Results of posthoc test (after LME) testing the impact of location and depth on 23S rRNA gene, *cbbL* gene and *bchY* gene observed richness (S) and alpha diversity (Shannon index). Cou = Counozouls, Männ = Männikjärve, Siik = Siikaneva. Abi = Abisko, D1 = 0 - 5 cm, D2 = 5 - 10 cm, D3 = 10 - 15 cm. Significant *p-values* in bold.

		Location						Depth Cou			Depth Män			Depth Siik			Depth Abi		
		Cou - Män	Cou - Siik	Cou - Abi	Män - Siik	Män - Abi	Abi - Siik	**D1 - D2**	**D1 - D3**	**D2 - D3**	**D1 - D2**	**D1 - D3**	**D2 - D3**	**D1 - D2**	**D1 - D3**	**D2 - D3**	**D1 - D2**	**D1 - D3**	**D2 - D3**
23S rRNA gene (oxygenic phototrophs)	S	**0.029**	0.53	**0.011**	0.28	0.95	0.12	**0.017**	**0.036**	0.86	0.94	**<0.001**	**<0.001**	0.69	0.25	0.63	0.98	0.8	0.71
	Shannon	0.82	0.62	0.8	0.98	0.33	0.19	**0.023**	**0.021**	0.99	0.68	**0.006**	**0.002**	0.79	0.2	0.45	0.78	0.36	0.74
cbbL gene (chemoautotrophs)	S	0.48	**<0.001**	0.24	**0.006**	0.95	**0.015**	0.61	**0.005**	**0.012**	0.094	**<0.001**	**<0.001**	0.82	0.12	**0.05**	0.071	**<0.001**	**<0.001**
	Shannon	0.97	**0.036**	0.3	0.081	0.54	0.57	0.26	**0.009**	0.11	**0.02**	**<0.001**	**<0.001**	0.77	0.1	0.27	0.55	**<0.001**	**<0.001**
bchY gene (AAnPBs)	S	0.43	0.99	0.65	0.3	0.074	0.8	0.99	0.33	0.4	0.99	**<0.001**	**<0.001**	0.24	0.84	0.11	0.21	**0.028**	0.39
	Shannon	**0.003**	**0.021**	0.94	0.69	**0.009**	0.058	0.29	0.051	0.48	**0.013**	0.71	**0.04**	**0.002**	**0.006**	0.72	0.78	0.12	0.31

Supplementary Table. 8| Primer pairs used in this study.

Analysis	Microorganism targeted	Targeted gene	Primers	Amplicon Size	Primer sequence	Reference
(a) PCR	Prokaryotes	16S rRNA	PCR1-515F	412 bp	5’ GTG YCA GCM GCC GCG GTA 3’	1
			PCR1-909R		5’ CCC CGY CAA TTC MTT TRA GT 3’	
	Photosynthetic microorganisms	23S rRNA	P23SrV_f1	410 bp	5’ GGA CAG AAA GAC CCT ATG AA 3’	2
			P23SrV_r1		5’ CAG CCT GTT ATC CCT AGA G 3’	
	Chemoautotrophs	*cbbL*	cbbL-IA-CHEM	479 bp	5’ GAR GGN TCN GTN GTY AAC GT 3’	3
			cbbL-IA-r		5’ GTA RTC GTG CAT GAT GAT SGG 3’	
	AAnPB	*bchY*	bchY-fwd	500 bp	5’ CCN CAR CAN ATG TGY CCN GCN TTY GG 3’	4
			bchY-rev		5’ GGR TCN RCN GGR AAN ATY TCN CC 3’	
(b) ddPCR	Prokaryotes	16S rRNA	L/Prba338f	180 bp	5′- ACT CCT ACG GGA GGC AGC AG -3′	5
			K/Prun518r		5′- ATT ACC GCG GCT GCT GG -3′	
	Cyanobacteria	16S rRNA	16SCF	164 bp	5'-GGC AGC AGT GGG GAA TTT TC-3'	6
			16SUR		5'-GTM TTA CCG CGG CTG CTG G-3'	
	Photosynthetic microorganisms	23S rRNA	23S255f	160 bp	5’- GGA TTA GAT ACC CYD GTA GTC C -3’	7
			P23SrV_r1		5’ – TCA GCC TGT TAT CCC TAG AG -3’	2
	Chemoautotrophs	*cbbL*	cbbLR1F	274 bp	5’- AAG GAY GAC GAG AAC ATC -3’	8
			cbbLRintR		5’- TGC AGS ATC ATG TCR TT -3’	
	AAnPB	*pufM*	pufM forward 557	193 bp	5’-TAC GGS AAC CTG TWC TAC-3’	9
			pufM reverse 750		5’-CCA TSG TCC AGC GCC AGA A-3’	

Supplementary Table. 9| PCR reaction conditions used in this study.

Targeted gene	16S rRNA	23S rRNA	*bchY*	*cbbL*
Annealing temperature	55˚C	55˚C	50˚C	52˚C
PCR conditions	95°C – 10 min<br><br>35 cycles:<br>94°C – 60 sec<br>55°C – 40 sec<br>72 ˚C – 30 sec<br><br>72°C – 10 min	95°C – 10 min<br><br>35 cycles:<br>94°C – 60 sec<br>55°C – 45 sec<br>72 ˚C – 45 sec<br><br>72°C – 10 min	95°C – 10 min<br><br>45 cycles:<br>94°C – 60 sec<br>50°C – 45 sec<br>72 ˚C – 60 sec<br><br>72°C – 10 min	95°C – 10 min<br><br>38 cycles:<br>94°C – 60 sec<br>52°C – 30 sec<br>72 ˚C – 60 sec<br><br>72°C – 10 min

Supplementary Table. 10| Reaction conditions for ddPCR.

Primers	L/Prba338f/<br>K/Prun518r	16SCF/<br>16SUR	23S255f/<br>P23Srv-r1	cbbLR1F/<br>cbbLRintR1	pufMfwd557/<br>pufMrev750
Primer concentration	250 nM	150 nM	250 nM	250 nM	250 nM
DNA dilution	1/1 000	1/100	1/100 (D1, D2) and 1/10 (D3)	1/10	1/100 (D1, D2) and 1/10 (D3)
Annealing temperature	61˚C	52˚C	57.6˚C	53˚C	50.2˚C
PCR conditions	95°C – 5 min<br>40 cycles:<br>94°C – 30 sec<br>61°C – 30 sec<br>4°C – 5 min<br>98°C – 10 min<br><br>Ramp rate = 1°C/sec	95°C – 5 min<br>40 cycles:<br>94°C – 30 sec<br>52°C – 60 sec<br>4°C – 5 min<br>98°C – 10 min<br><br>Ramp rate = 2°C/sec	95°C – 5 min<br>40 cycles:<br>94°C – 30 sec<br>57.6°C – 60 sec<br>4°C – 5 min<br>98°C – 10 min<br><br>Ramp rate = 2°C/sec	95°C – 5min<br>45 cycles:<br>94°C – 60 sec<br>53°C – 2 min<br>4°C – 5 min<br>98°C – 10 min<br><br>Ramp rate = 1°C/sec	95°C – 5min<br>45 cycles:<br>94°C – 60 sec<br>50.2°C – 2 min<br>4°C – 5 min<br>98°C – 10 min<br><br>Ramp rate = 1°C/sec

Supplementary Table. 11| Description of RStudio packages and functions used in this study.

RStudio package	Functions	Description	Reference
Phyloseq package v1.44.0	*rarefy_even_depth*	Analysis of sequencing data	10
ggplot2 v3.5.1		Graphical representation	11
Igraph v1.4.3	*cluster_fast_greedy*	Network analysis and visualization	12
ggpubr v0.6.0	*stat_compare_means*	Mean comparison p-value in a *ggplot*	13
FactoMineR v2.11	*PCA*<br>*MFA*	Multivariate exploratory data analysis	14
vegan v2.6.4	*adonis2*<br>*decostand*<br>*metaMDS*<br>*varpart*	Community analysis in ecology	15
corrplot v0.92	*corrplot*	Visual exploratory tool on correlation matrix	16
Stats 4.4.1	*hclust*	All basics functions	17
nlme v3.1-164	*lme*	Linear and Non Linear Mixed Effects Models	18
jSDM v0.2.6	*JSDM_binomial_probit*<br>*get_enviro_cor*	Joint Species Distribution Models	19
Hypervolumes v3.1.4		Estimates shape and volume of data	20
spatialRF v1.1.4	*plot_importance*	Selection of spatial predictors for spatial regression with random forest	21

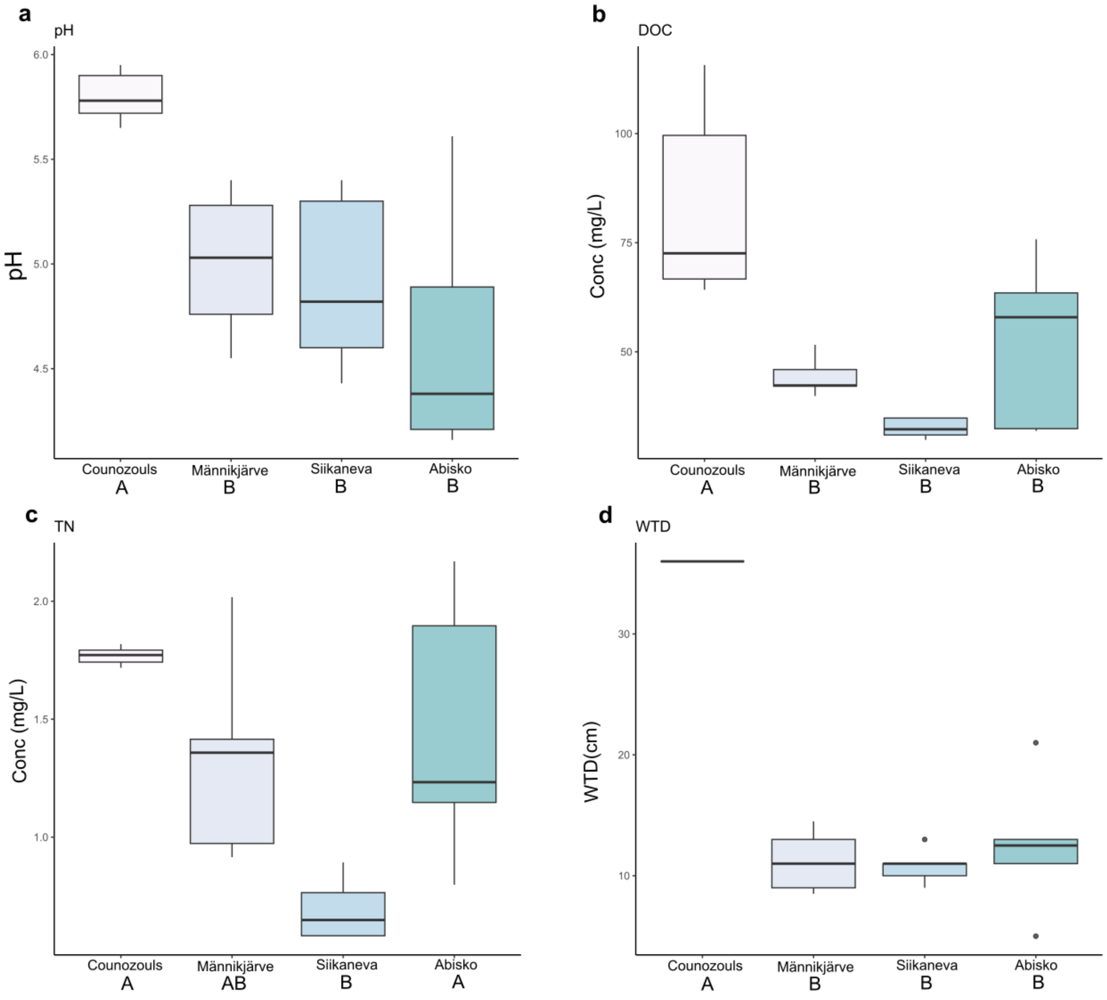

Supplementary Fig. 1| Boxplot describing (a) pH, (b) DOC, (c) TN and (d) WTD for each location. Uppercase letters represent the differences between each peatland. DOC = dissolved organic carbon, TN = total nitrogen and WTD = water table depth.

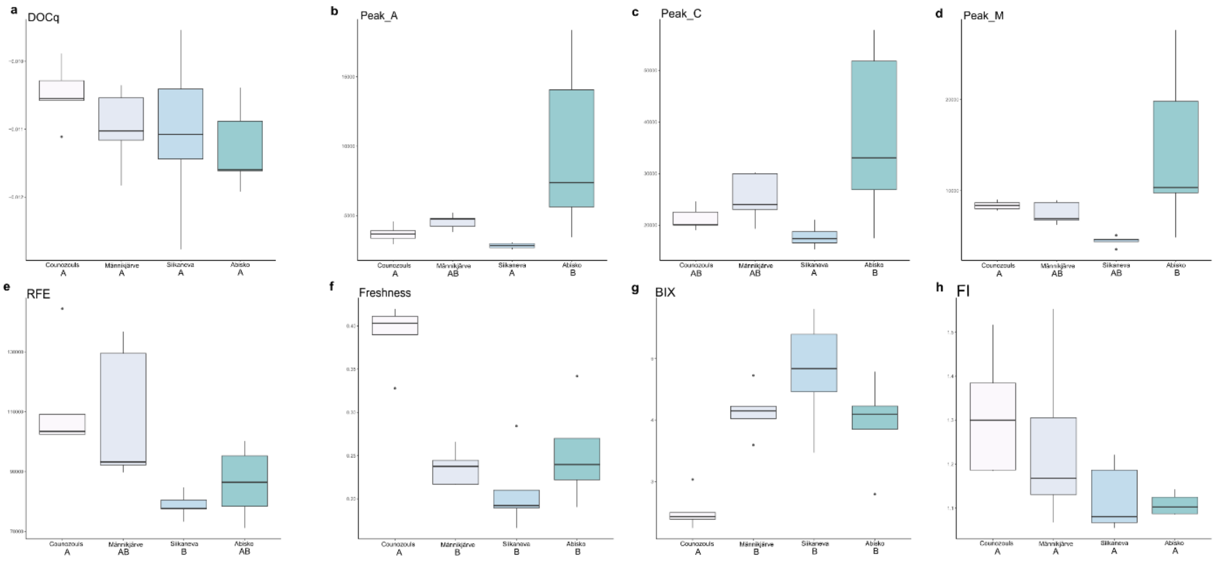

Supplementary Fig. 2| Boxplot describing organic matter quality index (DOCq, peak_A, peak_C, peak_M, RFE, freshness, BIX and FI) for each location. Uppercase letters represent the differences between each peatland. DOC = dissolved organic carbon, RFE = relative fluorescence efficiency, BIX = biological index and FI = fluorescence index.

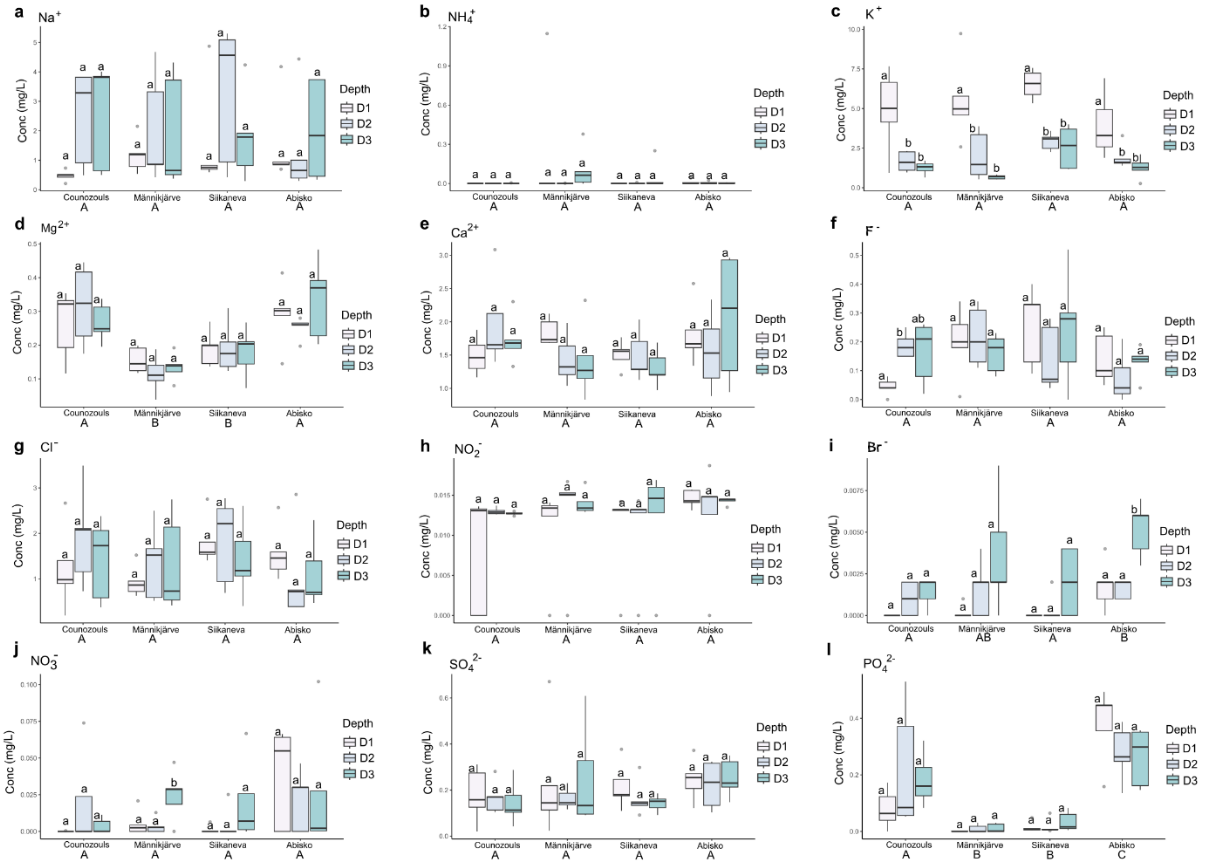

Supplementary Fig. 3| Boxplot describing cations (Na$^+$, NH$_4^+$, K$^+$, Mg^{2+} and Ca^{2+}) and anions (F$^-$, Cl$^-$, NO$_2$, NO$_3^-$, Br$^-$, SO$_4^{2-}$ and PO$_4^{3-}$) for each location. Uppercase letters represent the differences between each peatland, lowercase letters represent the differences

between depth (D1, D2 and D3) at each location. D1 = 0 - 5 cm, D2 = 5 - 10 cm and D3 = 10 - 15 cm.

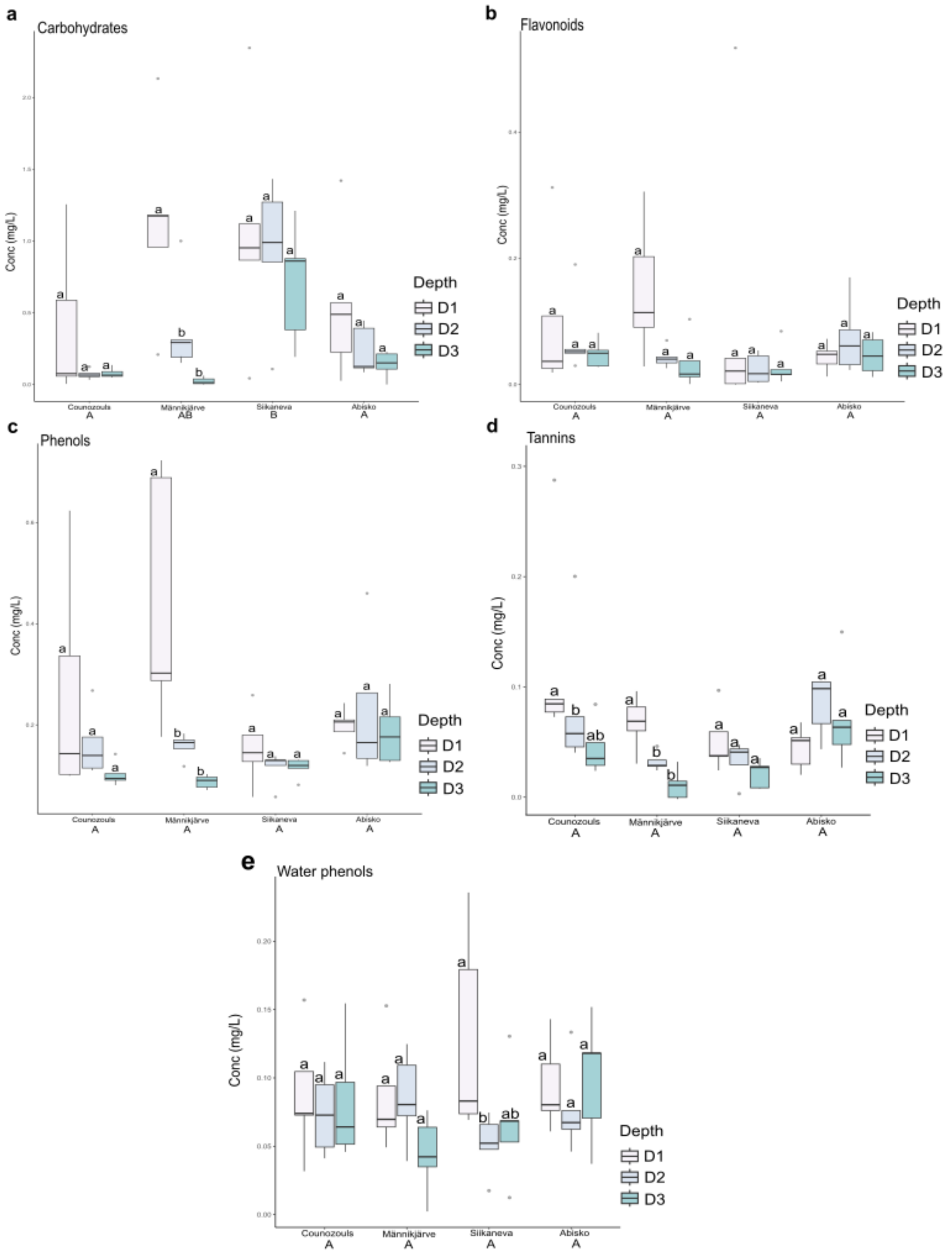

Supplementary Fig. 4| Boxplot describing metabolites (carbohydrates, flavonoids, phenols, tannins and water phenols) for each location. Uppercase letters represent the differences between each peatland, lowercase letters represent the differences between depth (D1, D2 and D3) at each location. D1 = 0 - 5 cm, D2 = 5 - 10 cm and D3 = 10 - 15 cm.

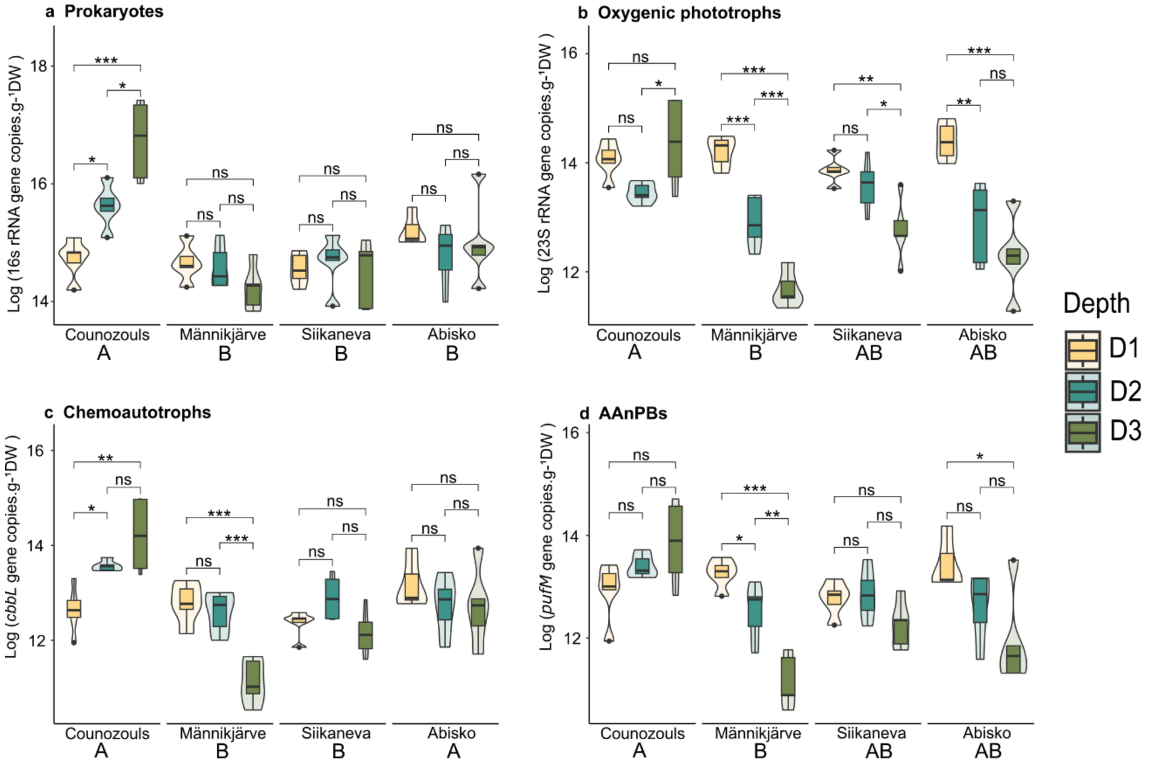

Supplementary Fig. 5| Absolute quantification using ddPCR. (a) 16S rRNA gene (prokaryotes), **(b)** 23S rRNA gene (oxygenic phototrophs), **(c)** *cbbL* (chemoautotrophs) and **(d)** *pufM* (AAnPBs) at different depths in the four peatlands. Violin plots are showing the data distribution shape while boxplots are representing the logarithm of the total gene copies.g^{-1} DW. D1 = 0 - 5 cm; D2 = 5 - 10 cm and D3 = 10 - 15 cm. ns = not significant, *: P < 0.05, **: P < 0.01 and ***: P < 0.001.

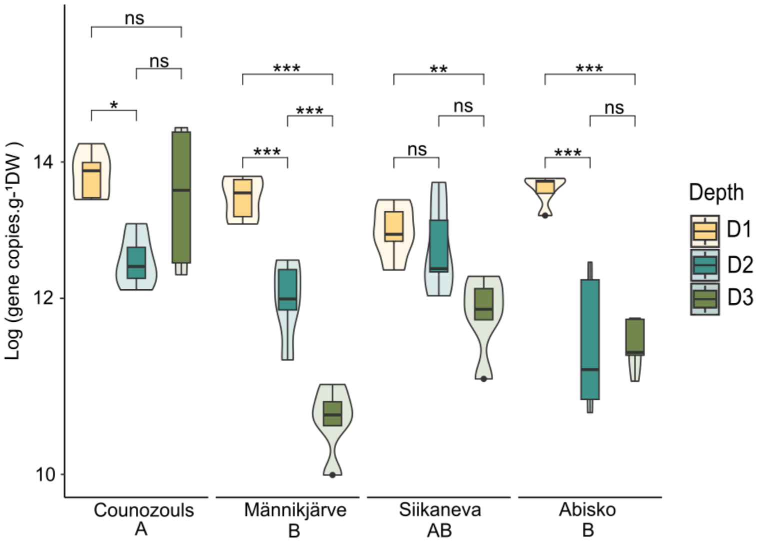

Supplementary Fig. 6| Absolute quantification of cyanobacteria using ddPCR. Violin plots are showing the data distribution shape while boxplots are representing the logarithm of the total gene copies.g^{-1} DW. D1 = 0 - 5 cm; D2 = 5 - 10 cm and D3 = 10 - 15 cm. ns = not significant, *: P < 0.05, **: P < 0.01 and ***: P < 0.001.

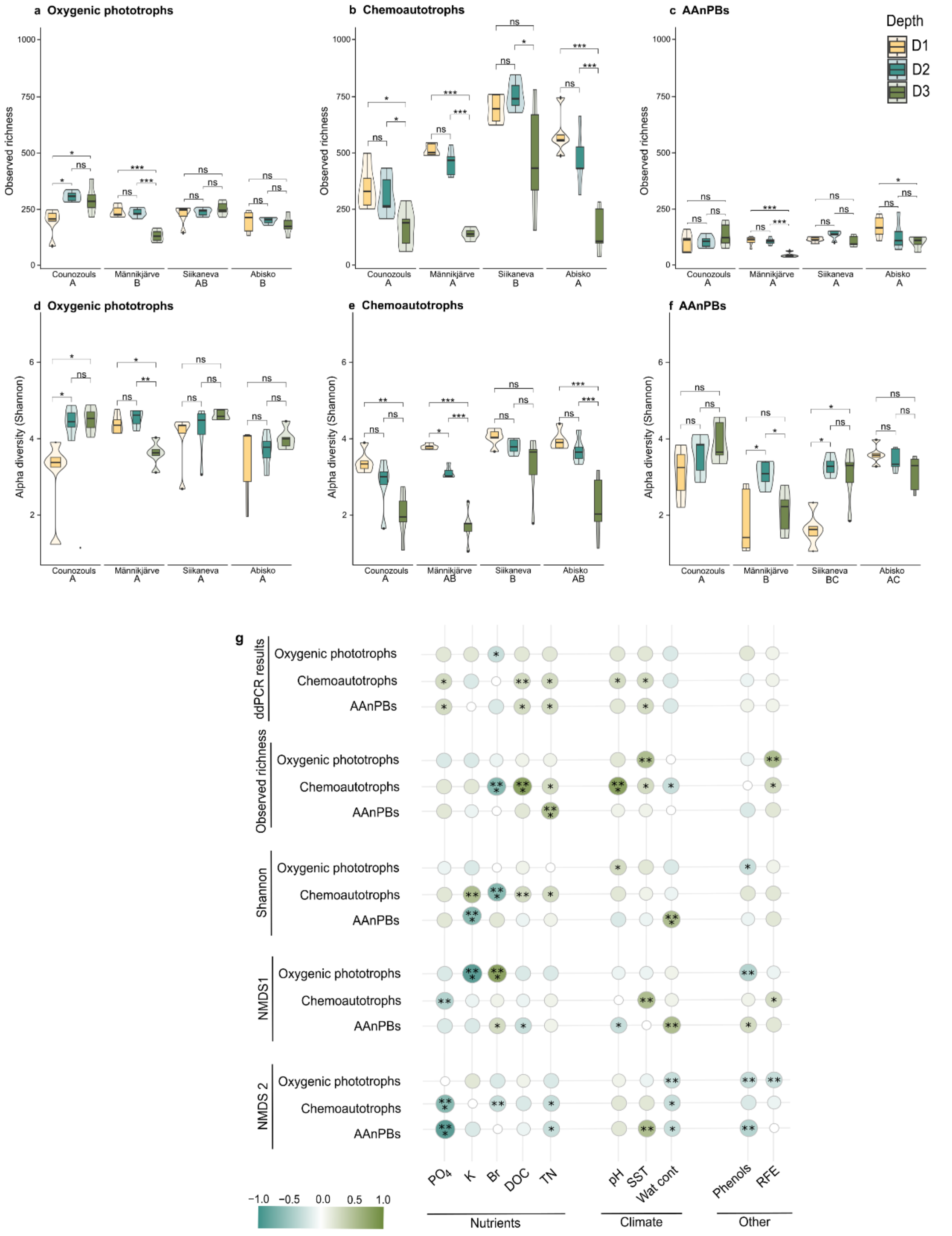

Supplementary Fig. 7| Diversity description of 23S rRNA, *cbbL* and *bchY* genes at each location and depth (D1, D2 and D3). (a), (b) and (c) Observed richness and **(d), (e) and (f)** Alpha diversity (Shannon index). Violin plots are showing the data distribution shape while boxplots are representing the logarithm of the total gene copies.g^{-1} DW. D1 = 0 - 5 cm, D2 = 5 - 10 cm and D3 = 10 - 15 cm. **(g)** Correlation plot (Pearson correlation) of CFMs community abundance (ddPCR results), observed richness, alpha-diversity (Shannon) and beta-diversity (NMDS1 and NMDS2) with nutrients (PO_4^{2-}, K^+, Br^-, DOC, TN), climate (pH, SST, wat cont), phenols and RFE. DOC = dissolved organic carbon; TN = total nitrogen; SST = spring soil temperature and wat cont = water content. ns = not significant, *: P < 0.05, **: P < 0.01 and ***: P < 0.001.

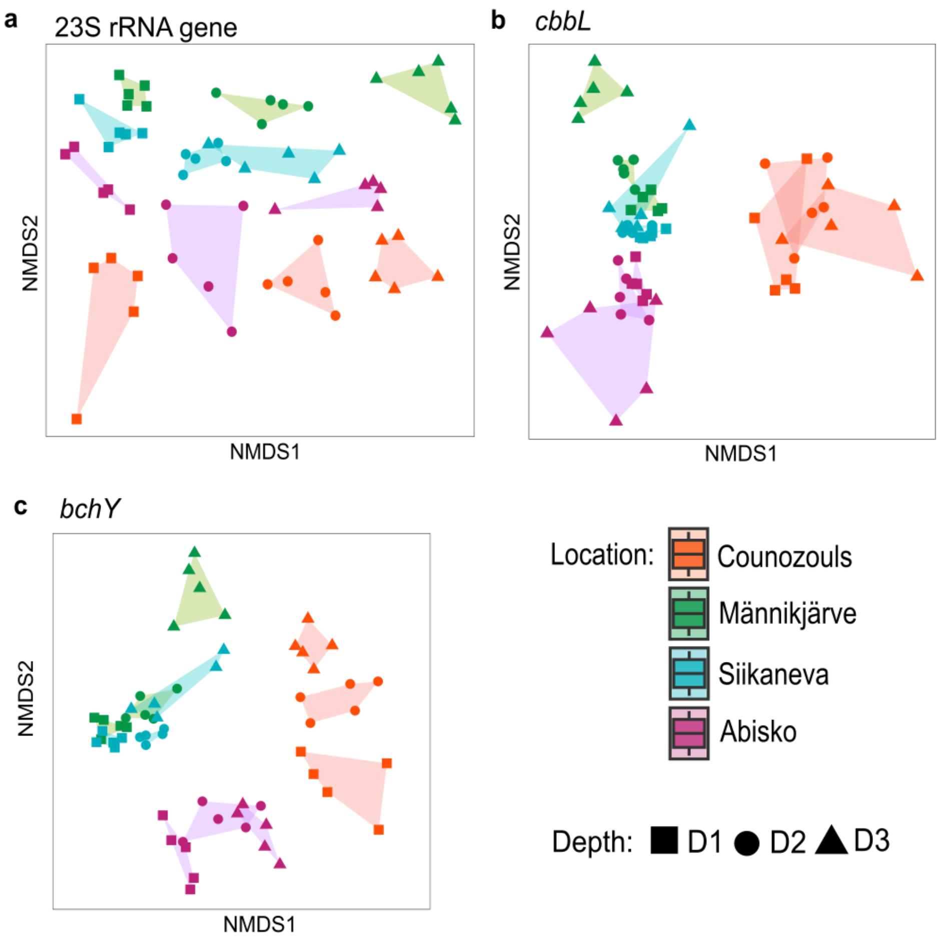

Supplementary Fig. 8| NMDS showing the community structure. **(a)** Oxygenic phototrophs (23S rRNA gene), **(b)** chemoautotrophs (*cbbL* gene) and **(c)** AAnPBs (*bchY* gene). The Bray-Curtis dissimilatory index has been used. D1 = 0 - 5 cm; D2 = 5 - 10 cm and D3 = 10 - 15 cm.

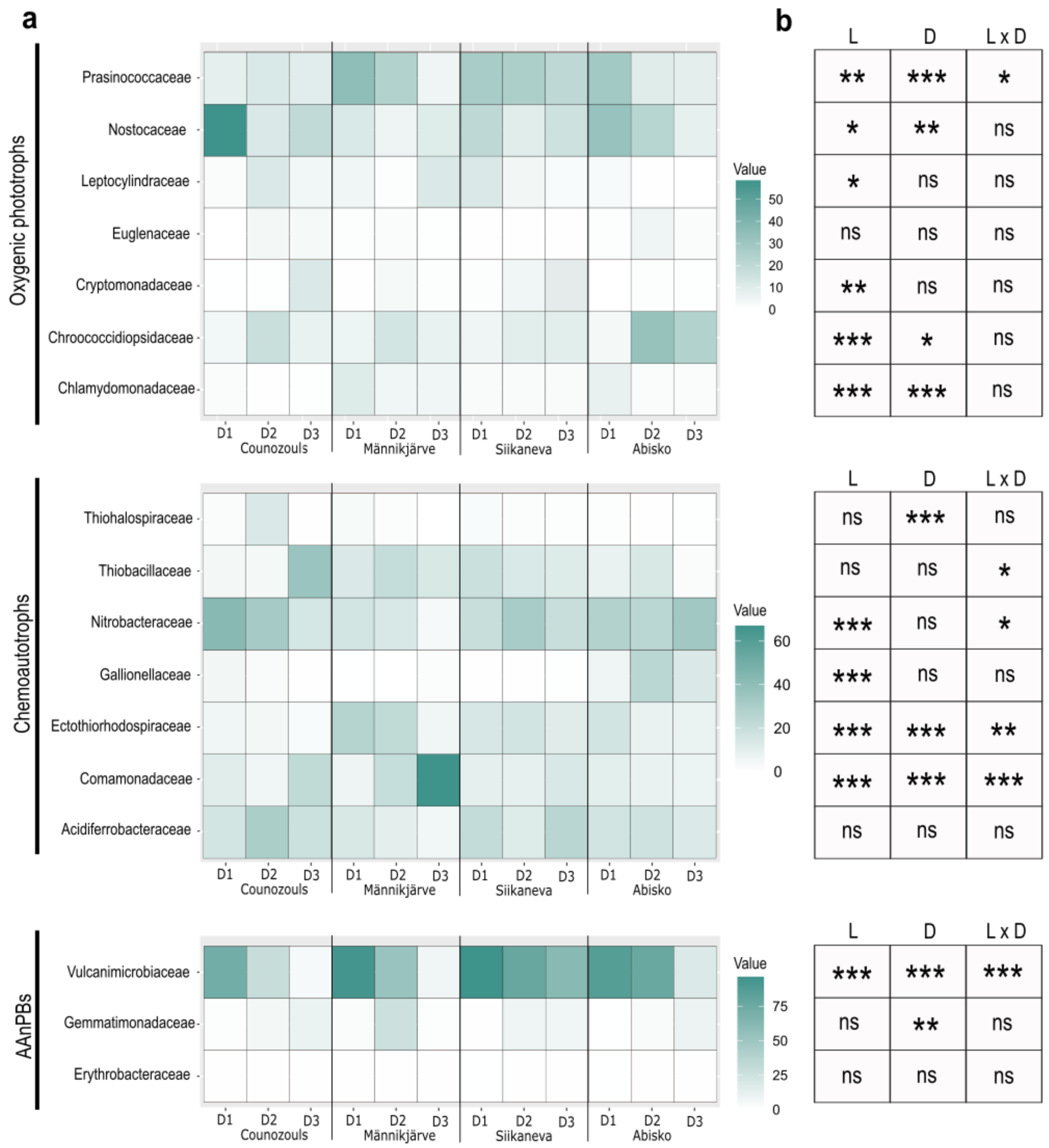

Supplementary Fig. 9| Impact of location and depth on relative abundance of ASVs aggregated by family. (a) Heatmaps showing the relative abundance of ASVs aggregated by family according to location and depth. Only family with abundance higher than 5% were kept. Light color represents low abundances while dark color represents higher abundances. D1 = 0 - 5 cm, D2 = 5 - 10 cm and D3 = 10 - 15 cm. **(b)** P-values of the explanatory power of location, depth and location with depth. L = location; D = depth. ns = not significant; *: P < 0.05, **: P < 0.01 and ***: P < 0.001.

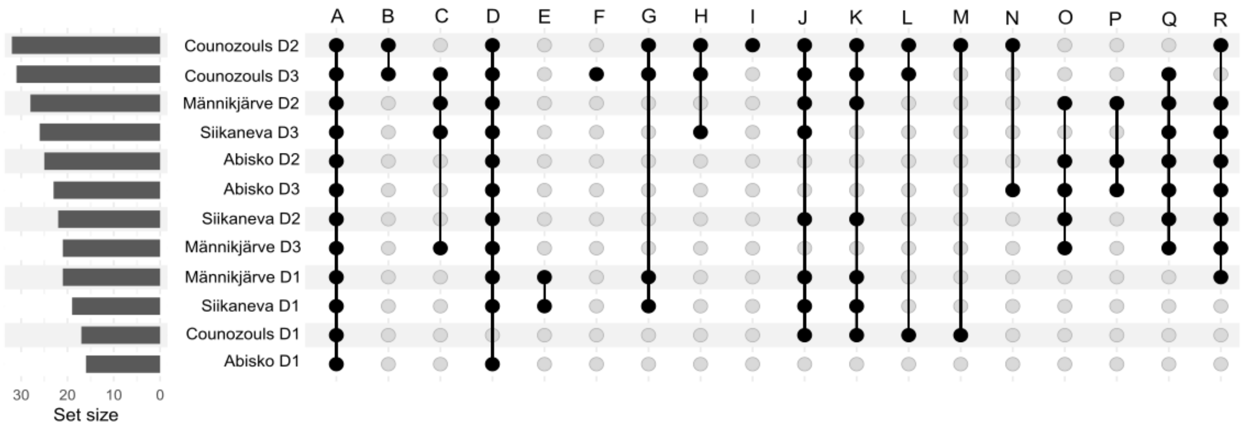

A	Bacillariophyceae, Synurophyceae, Trebouxiophyceae, Euglenophyceae, Mediophyceae, Palmophyllophyceae, Cryptophyceae, Cyanophyceae,
B	Anthocerotopsida, Myxococcia, Omnitrophia
C	Porphyridiophyceae
D	Firestonebacteria, Defferrisomatia
E	Glaucophyceae
F	Actinobacteria
G	Dinophyceae
H	Bacteroidia
I	Clostridia, Leiosporocerotopsida
J	Ulvophyceae
K	Conjugatophyceae
L	Xanthophyceae
M	Synergistia
N	Clostridia
O	Planctomycetes
P	Florideophyceae
Q	Porphyridiophyceae
R	Desulfuromonodales

Supplementary Fig. 10| Upset plot of the presence of ASVs aggregated by class for the 23S rRNA gene in the four peatland sites. D1 = 0 - 5 cm, D2 = 5 - 10 cm and D3 = 10 - 15 cm.

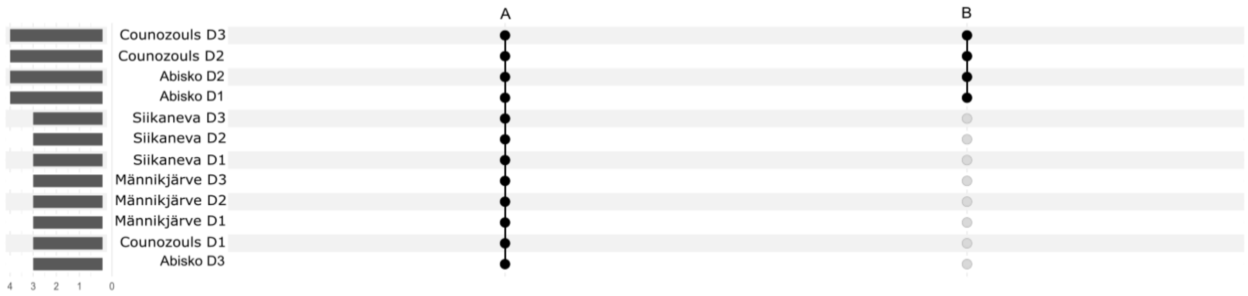

A	Alphaproteobacteria, Betaproteobacteria, Gammaproteobacteria
B	unassigned

Supplementary Fig. 11| Upset plot of the presence of ASVs aggregated by class for the *cbbL* gene in the four peatland sites. D1 = 0 - 5 cm, D2 = 5 - 10 cm and D3 = 10 - 15 cm.

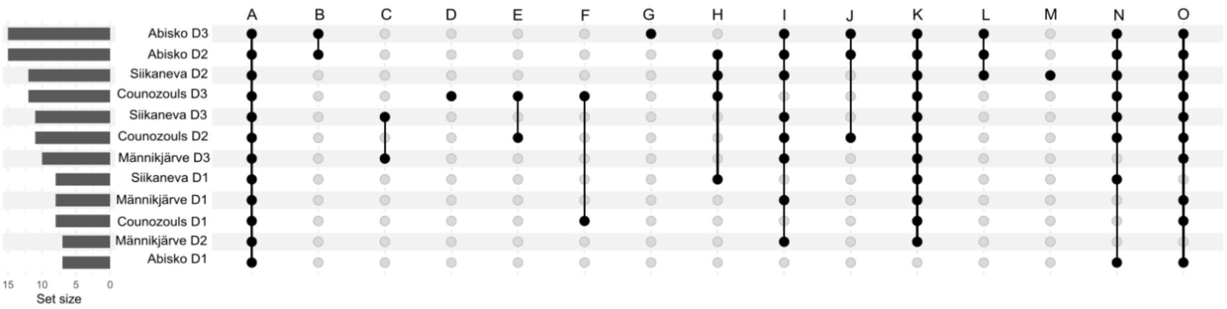

A	Vulcanimicrobiia, unassigned, Gemmatimonadetes, Terriglobia, Alphaproteobacteria
B	Chitinophagia, Thermoleophilia, Vicinamibacteria
C	Deinococcota, Desulfovibrionia
D	Longimicrobiia
E	Deltaproteobacteria
F	Chloroflexia
G	Desulfobacteria
H	Desulfuromonadia
I	Actinomycetota
J	Acidimicrobiia
K	Betaproteobacteria
L	Actinomycetes
M	Myxococcia
N	Gammaproteobacteria
O	Planctomycetia

Supplementary Fig. 12| Upset plot of the presence of ASVs aggregated by class for the *bchY* gene in the four peatland sites. D1 = 0 - 5 cm, D2 = 5 - 10 cm and D3 = 10 - 15 cm.

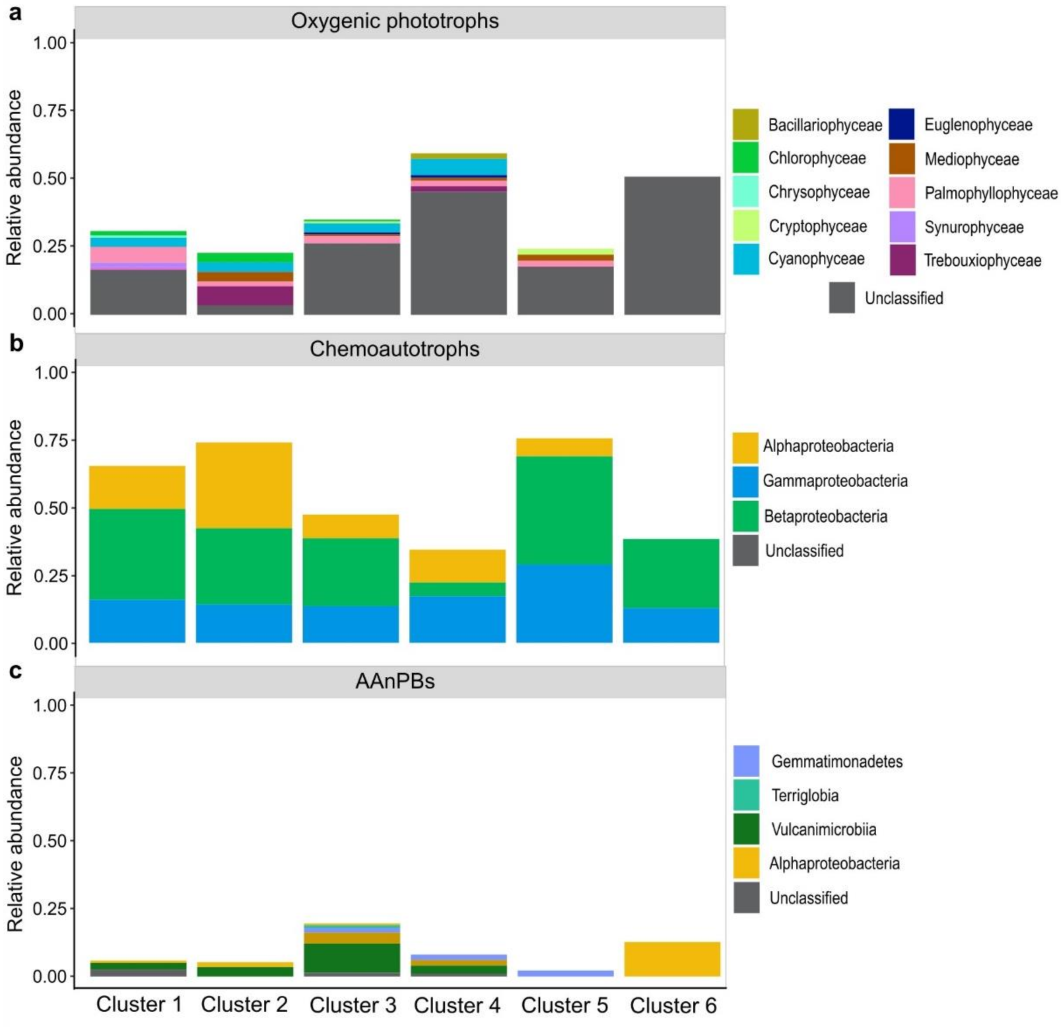

Supplementary Fig. 13| Barplot of the relative abundance of each class constituting the different clusters. **(a)** oxygenic phototrophs, **(b)** chemoautotrophs and **(c)** AAnPBs.

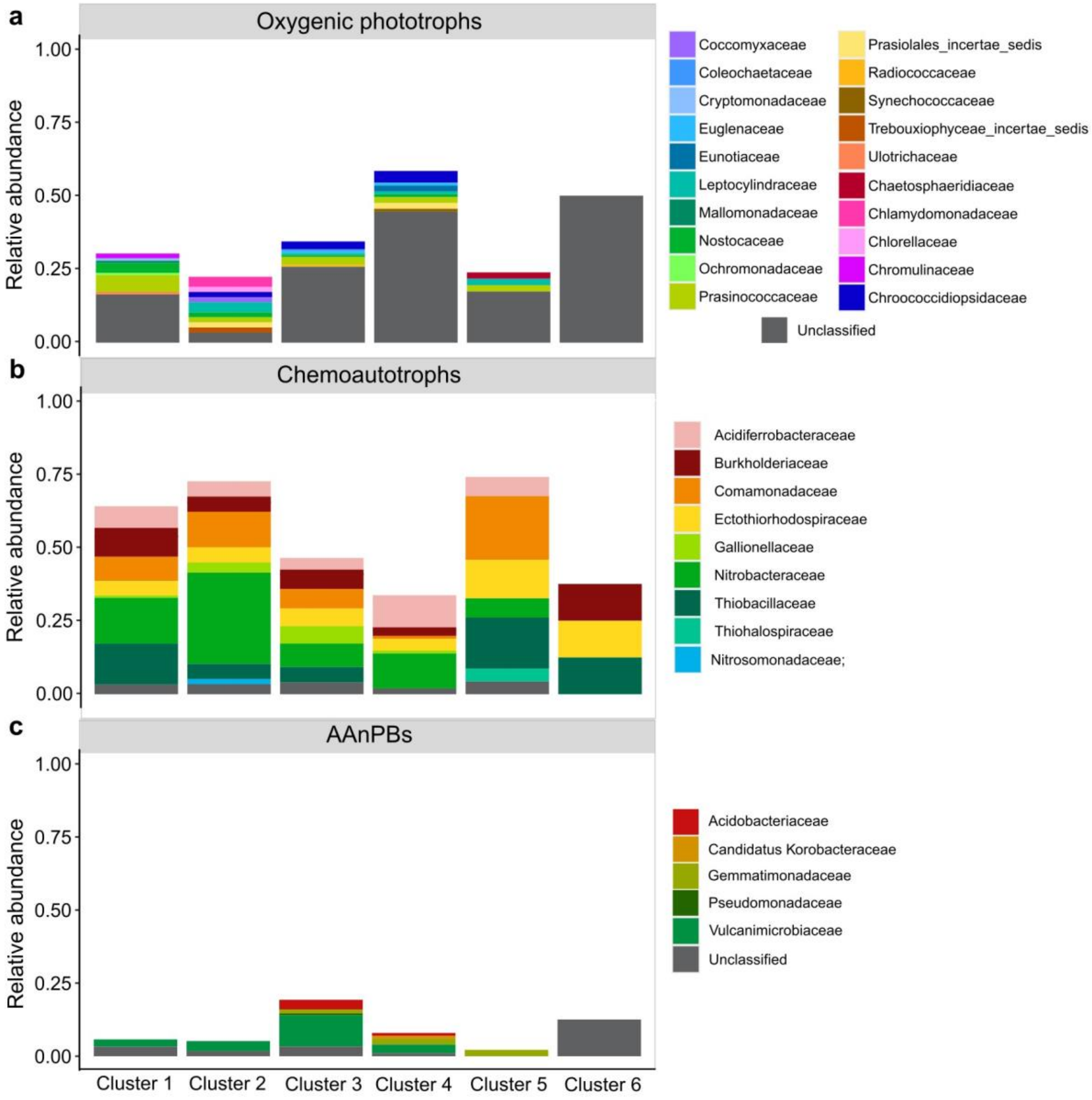

Supplementary Fig. 14| Barplot of the relative abundance of each family constituting the different clusters. **(a)** oxygenic phototrophs, **(b)** chemoautotrophs and **(c)** AAnPBs.

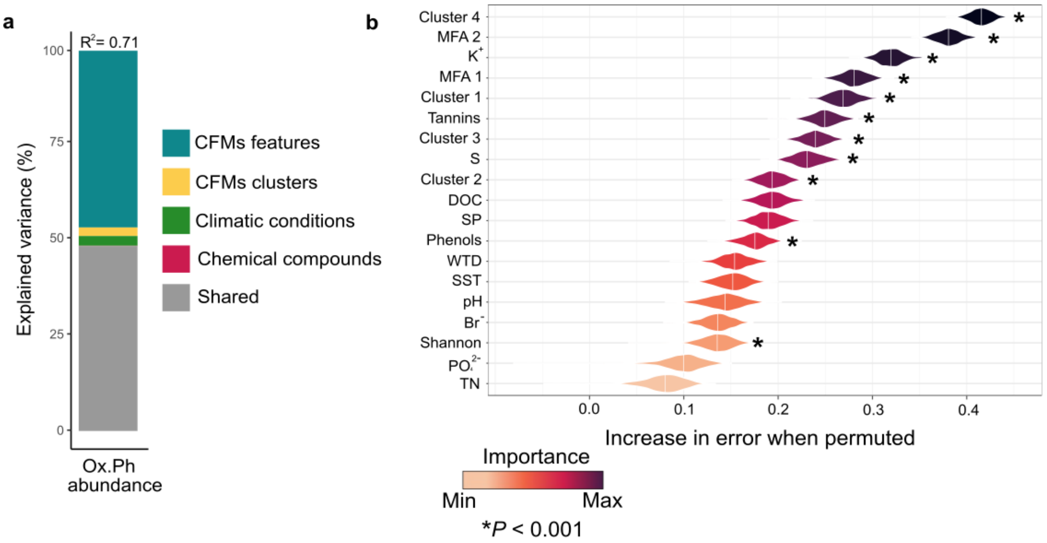

Supplementary Fig. 15| Drivers of absolute quantification of oxygenic phototrophs in peatlands. (a) Variation partitioning modelling evaluating the unique and shared portions of variation in oxygenic phototrophs abundance. CFMs features refer to ASV richness (S), alpha diversity (Shannon) and community composition (MFA axis 1 and 2); CFMs clusters refers to relative abundance of species from clusters 1 to 4 generated by the JSDM model; Climatic conditions refer to WTD, SP and SST; Chemical compounds refer to tannins, phenols, DOC, K^+, pH, TN, PO_4^{2-} and Br^- and Shared refers to the percentage of shared variation explained by all predictors. **(b)** Results from random forest analysis showing the relative importance of the different drivers of the absolute quantification of oxygenic phototrophs in peatlands. Ox.Ph = oxygenic phototrophs; WTD = water table depth; SP = spring precipitation; SST = spring soil temperature; DOC = dissolved organic carbon; TN = total nitrogen. *: P < 0.001.

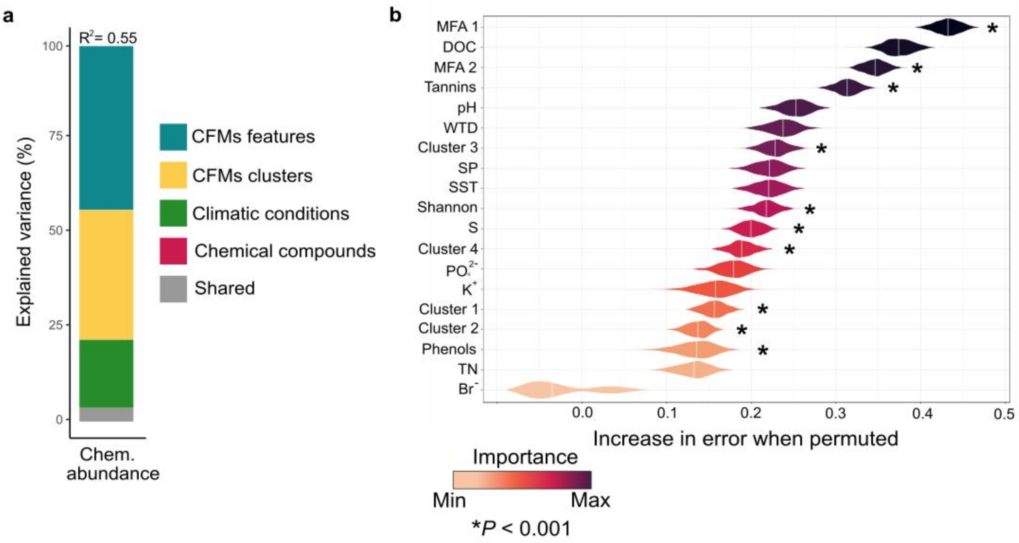

Supplementary Fig. 16| Drivers of absolute quantification of chemoautotrophs in peatlands. (a) Variation partitioning modelling evaluating the unique and shared portions of variation in chemoautotrophs abundance. CFMs features refer to ASV richness (S), alpha diversity (Shannon) and community composition (MFA axis 1 and 2); CFMs clusters refers to relative abundance of species from clusters 1 to 4 generated by the JSDM model; Climatic conditions refer to WTD, SP and SST; Chemical compounds refer to tannins, phenols, DOC, K^+, pH, TN, PO_4^{2-} and Br^- and Shared refers to the percentage of shared variation explained

by all predictors. **(b)** Results from random forest analysis showing the relative importance of the different drivers of the absolute quantification of chemoautotrophs in peatlands. Chem. = chemoautotrophs; WTD = water table depth; SP = spring precipitation; SST = spring soil temperature; DOC = dissolved organic carbon; TN = total nitrogen. *: P < 0.001.

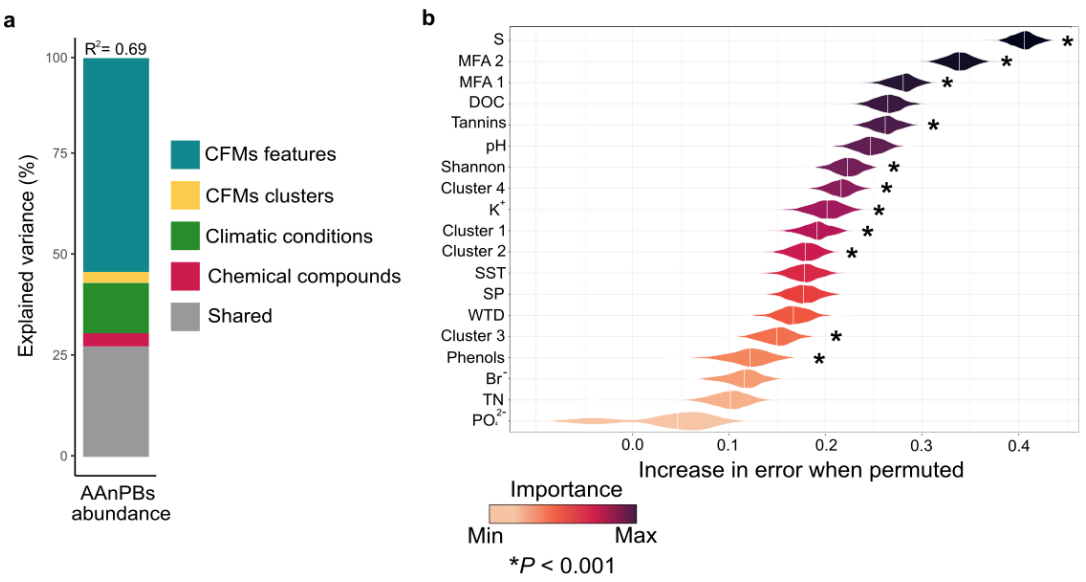

Supplementary Fig. 17| Drivers of absolute quantification of AAnPBs in peatlands. (a) Variation partitioning modelling evaluating the unique and shared portions of variation in AAnPBs abundance. CFMs features refer to ASV richness (S), alpha diversity (Shannon) and community composition (MFA axis 1 and 2); CFMs clusters refers to relative abundance of species from clusters 1 to 4 generated by the JSDM model; Climatic conditions refer to WTD, SP and SST; Chemical compounds refer to tannins, phenols, DOC, K^+, pH, TN, PO_4^{2-} and Br^- and Shared refers to the percentage of shared variation explained by all predictors. **(b)** Results from random forest analysis showing the relative importance of the different drivers of the absolute quantification of AAnPBs in peatlands. WTD = water table depth; SP = spring precipitation; SST = spring soil temperature; DOC = dissolved organic carbon; TN = total nitrogen. *: P < 0.001.

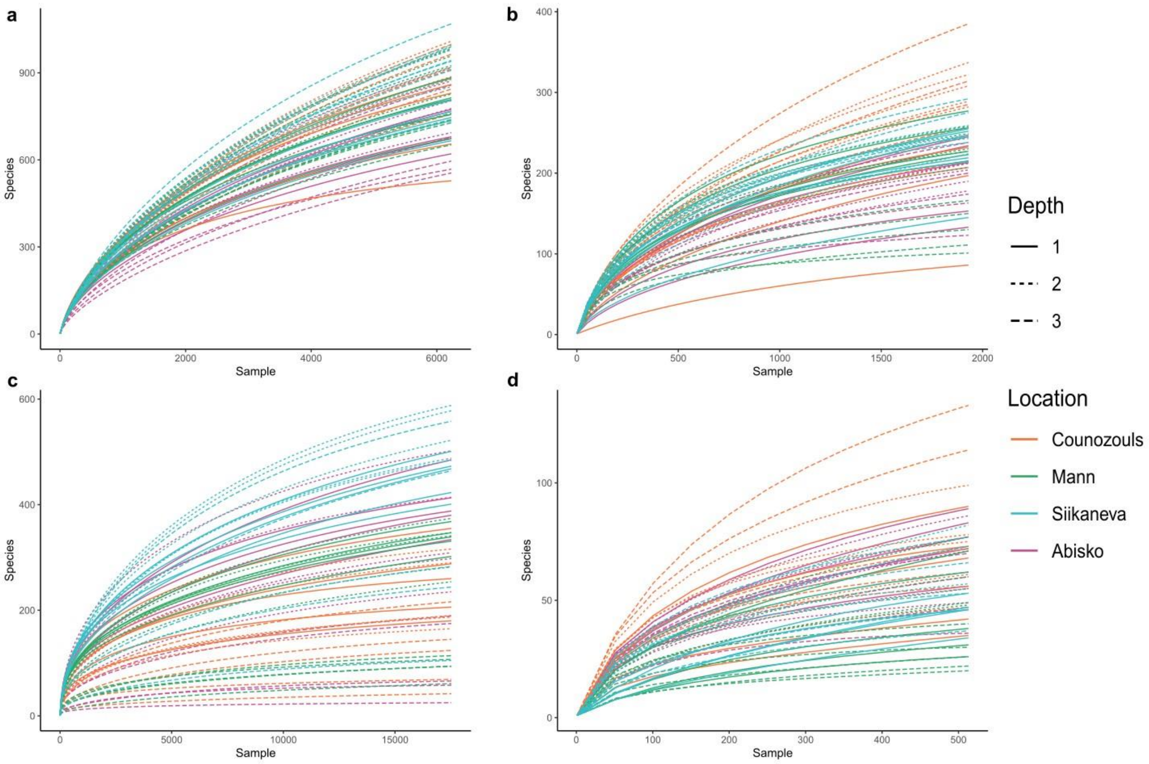

Supplementary Fig. 18| Rarefaction curves of microbial sequences after normalization of the number of sequences. **(a)** 16S rRNA gene, **(b)** 23S rRNA gene, **(c)** *cbbL* gene and **(d)** *bchY* gene.

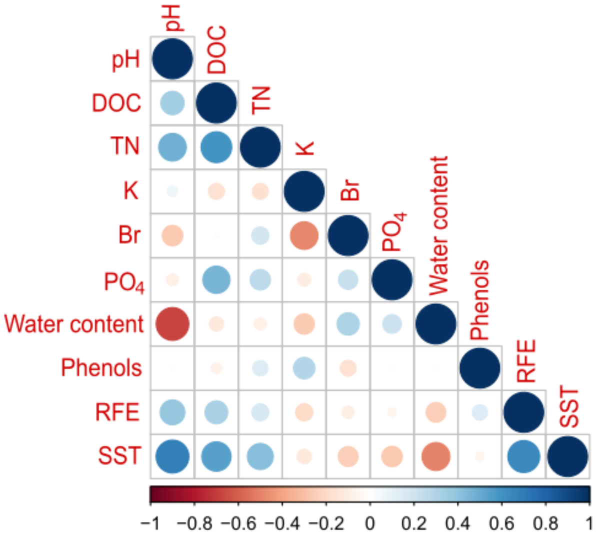

Supplementary Fig. 19| Correlation plot of environmental variables retained for principal component analysis (PCA). DOC = dissolved organic carbon, TN = total nitrogen, RFE = relative fluorescence efficiency and SST = spring soil temperature.

References

1. Wang, Y. & Qian, P. Y. Conservative Fragments in Bacterial 16S rRNA Genes and Primer Design for 16S Ribosomal DNA Amplicons in Metagenomic Studies. *PLoS One* **4**, e7401 (2009).
2. Sherwood, A. R. & Presting, G. G. Universal primers amplify a 23S rDNA plastid marker in Eukaryotic algae and cyanobacteria. *J Phycol* **43**, 605–608 (2007).
3. Alfreider, A. & Bogensperger, T. Specific detection of form IA RubisCO genes in chemoautotrophic bacteria. *J Basic Microbiol* **58**, 712 (2018).
4. Yutin, N. *et al.* BchY-based degenerate primers target all types of anoxygenic photosynthetic bacteria in a single PCR. *Appl Environ Microbiol* **75**, 7556–7559 (2009).
5. Øvreås, L., Forney, L., Daae, F. L. & Torsvik, V. Distribution of bacterioplankton in meromictic Lake Saelenvannet, as determined by denaturing gradient gel electrophoresis of PCR-amplified gene fragments coding for 16S rRNA. *Appl Environ Microbiol* **63**, 3367 (1997).
6. Oh, K. H., Jeong, D. H., Shin, S. H. & Cho, Y. C. Simultaneous quantification of cyanobacteria and Microcystis spp. using real-time PCR. *J Microbiol Biotechnol* **22**, 248–255 (2012).
7. Le Geay, M., Mayers, K., Küttim, M., Lauga, B. & Jassey, V. E. J. Development of a digital droplet PCR approach for the quantification of soil micro-organisms involved in atmospheric CO2 fixation. *Environ Microbiol* **26**, (2024).
8. Selesi, D., Pattis, I., Schmid, M., Kandeler, E. & Hartmann, A. Quantification of bacterial RubisCO genes in soils by cbbL targeted real-time PCR. *J Microbiol Methods* **69**, 497–503 (2007).
9. Du, H., Jiao, N., Hu, Y. & Zeng, Y. Real-time PCR for quantification of aerobic anoxygenic phototrophic bacteria based on pufM gene in marine environment. *J Exp Mar Biol Ecol* **329**, 113–121 (2006).
10. McMurdie, P. J. & Holmes, S. Phyloseq: An R Package for Reproducible Interactive Analysis and Graphics of Microbiome Census Data. *PLoS One* **8**, (2013).
11. Wickham, H. ggplot2: Elegant Graphics for Data Analysis. Springer-Verlag New York ISBN 978-3-319-24277-4. *https://ggplot2.tidyverse.org* (2016).
12. Csárdi, G. *et al.* igraph for R: R interface of the igraph library for graph theory and network analysis. Preprint at https://doi.org/10.5281/zenodo.14736815 (2025).
13. Kassambara, A. ggpubr: 'ggplot2' Based Publication Ready Plots. R package version 0.6.0, https://rpkgs.datanovia.com/ggpubr/.(2023).
14. Le, S., Mazet, J., Julie, J. & Husson, F. FactoMineR: an R package dedicated to multivariate Exploratory Data Analysis. vol. 25 (2008).
15. Oksanen J, Simpson G, Blanchet F, Kindt R, Legendre P, Minchin P, O'Hara R, Solymos P, Stevens M, Szoecs E, Wagner H, Barbour M, Bedward M, Bolker B, Borcard D, Borman T, Carvalho G, Chirico M, De Caceres M, Durand S, Evangelista H, FitzJohn R, Friendly M, Furneaux B, Hannigan G, Hill M, Lahti L, McGlinn D, Ouellette M, Ribeiro Cunha E, Smith T, Stier A, Ter Braak C, Weedon J (2025). vegan: Community Ecology Package. R package version 2.7-0, https://vegandevs.github.io/vegan/.
16. Wei T, Simko V (2024). R package 'corrplot': Visualization of a Correlation Matrix. (Version 0.95), https://github.com/taiyun/corrplot. *https://cran.r-project.org/web/packages/corrplot/citation.html*.

17. R Core Team. R: A language and environment for statistical computing. *R: A language and environment for statistical ## computing. R Foundation for Statistical Computing, Vienna, Austria*. Preprint at https://intro2r.com/citing-r.html (2022).
18. Pinheiro, J. C. & Bates, D. M. *Mixed-Effects Models in S and S-PLUS*. *Mixed-Effects Models in S and S-PLUS* (Springer-Verlag, 2000). doi:10.1007/b98882.
19. Warton, D. I. *et al.* So Many Variables: Joint Modeling in Community Ecology. *Trends in Ecology and Evolution* vol. 30 766–779 Preprint at https://doi.org/10.1016/j.tree.2015.09.007 (2015).
20. Blonder B, Morrow wcfCB, Brown S, Butruille G, Chen D, Laini A, Harris DJ, Violet C (2025). hypervolume: High Dimensional Geometry, Set Operations, Projection, and Inference Using Kernel Density Estimation, Support Vector Machines, and Convex Hulls. R package version 3.1.5, https://github.com/bblonder/hypervolume. *https://bblonder.r-universe.dev/hypervolume*.
21. Benito, B. M. spatialRF: easy spatial regression with random forest measurement. Zenodo. https://doi.org/10.5281/zenodo.4745208 (2021). *Journal of Statistical Software*.
